# New Antimicrobial Cyclodepsipeptides from a Freshwater Fungus from the Sierra Madre Oriental in Mexico

**DOI:** 10.1021/acsomega.4c10990

**Published:** 2025-01-29

**Authors:** Itzel Rubí Yeverino, Tania Paola Bocanegra Sosa, Laura Aguilar-Vega, Rodolfo García-Contreras, José L. Magaña-González, Mario Figueroa

**Affiliations:** † Departamento de Farmacia, Facultad de Química, Universidad Nacional Autónoma de México, Ciudad de México 04510, Mexico; ‡ Departamento de Microbiología y Parasitología, Facultad de Medicina, Universidad Nacional Autónoma de México, Ciudad de México 04510, Mexico; § Instituto Nacional de Psiquiatría “Ramón de la Fuente Muñiz”, Ciudad de México 14370, Mexico

## Abstract

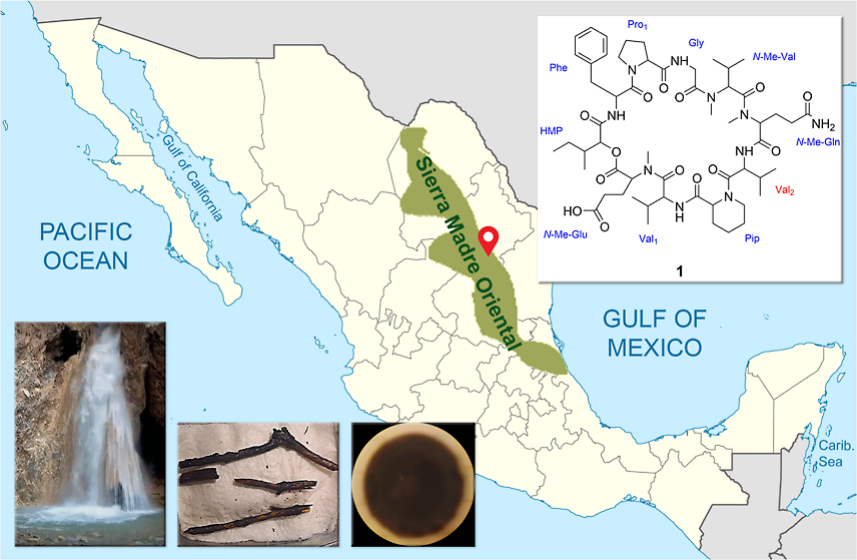

The Sierra Madre Oriental (SMO) in Mexico is a complex, unexplored geological area with multiple habitats and unique physical, chemical, and biological features. A bioactive-guided study of the organic extract from a solid-state fermentation culture from a taxonomically unidentified fungus isolated from submerged wood in the waterfall “El Caracol”, Nuevo Leon, at the SMO, led to the identification of three new cyclodepsipeptides (**1**–**3**) and the known Sch 217048 (**4**) and Sch 378161 (**5**). Structures of all compounds were elucidated by spectroscopic and spectrometric methods. The isolated compounds **4** and **5** showed antimicrobial activity against Gram-positive strains and the Gram-negative *Acinetobacter baumannii* ATCC 17978, including multidrug-resistant clinical strain A564. In addition, the compounds showed no toxic activity in the *Galleria mellonella* larvae model. Finally, the molecular networking analysis allowed us to annotate all the cyclodepsipeptides in the network. This is the first systematic chemical study of a fungus isolated from the SMO in Mexico.

## Introduction

Mountains cover 12% of the earth’s surface, 17% of Northern America, and 450,843 km^2^ of Mexico’s territory.^1−3^ One of Mexico’s most important mountain systems is the Sierra Madre Oriental (SMO), which represents Northern and Eastern Mexico’s most elevated mountain chain. Its length extends beyond 1000 km, and its average height is 2200 masl.^4^ The SMO is a complex geological area, possessing a wide variety of geographical features such as canyons, caverns, waterfalls, etc., which have diverse climatological conditions, ranging from cold to warm weather, with different insolation, moisture, and darkness levels, and runoffs that serve as a refuge for the mixed biota found there.^5,6^ Despite the numerous studies on the flora and fauna from the SMO, the number of microorganisms from this mountain system and its chemical studies remain little explored; macromycetes and endophytic fungi are the most studied microorganisms in this region.^7−10^

Fungi are one of the largest groups of microorganisms, with an estimated 2.2–3.8 million fungal species worldwide, and are the most prolific producers of a great diversity of bioactive secondary metabolites.^11^ Freshwater fungi (FWF) are one of the fungal groups that have been poorly studied both chemically and taxonomically.^12^ From the 3069–4145 FWF species described, around 300 metabolites have been isolated.^12,13^

**Figure 1:**
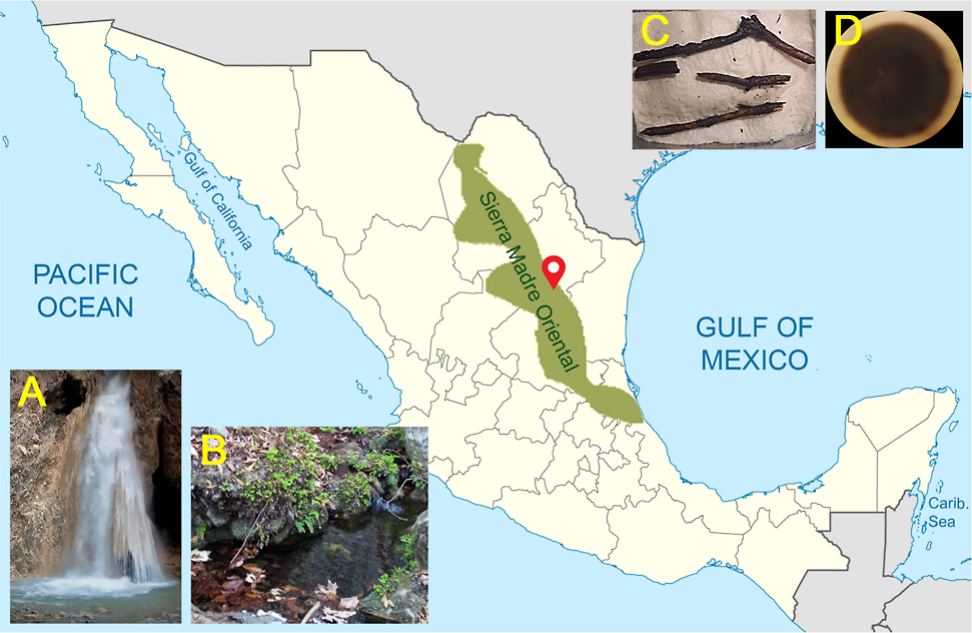
Collection site at the SMO in Iturbide, Nuevo Leon, Mexico: (A) waterfall “El Caracol” and (B) pond where the submerged wood samples were collected. (C) Decaying wood samples. (D) Fungus CR34 in PDA.

**Figure 2:**
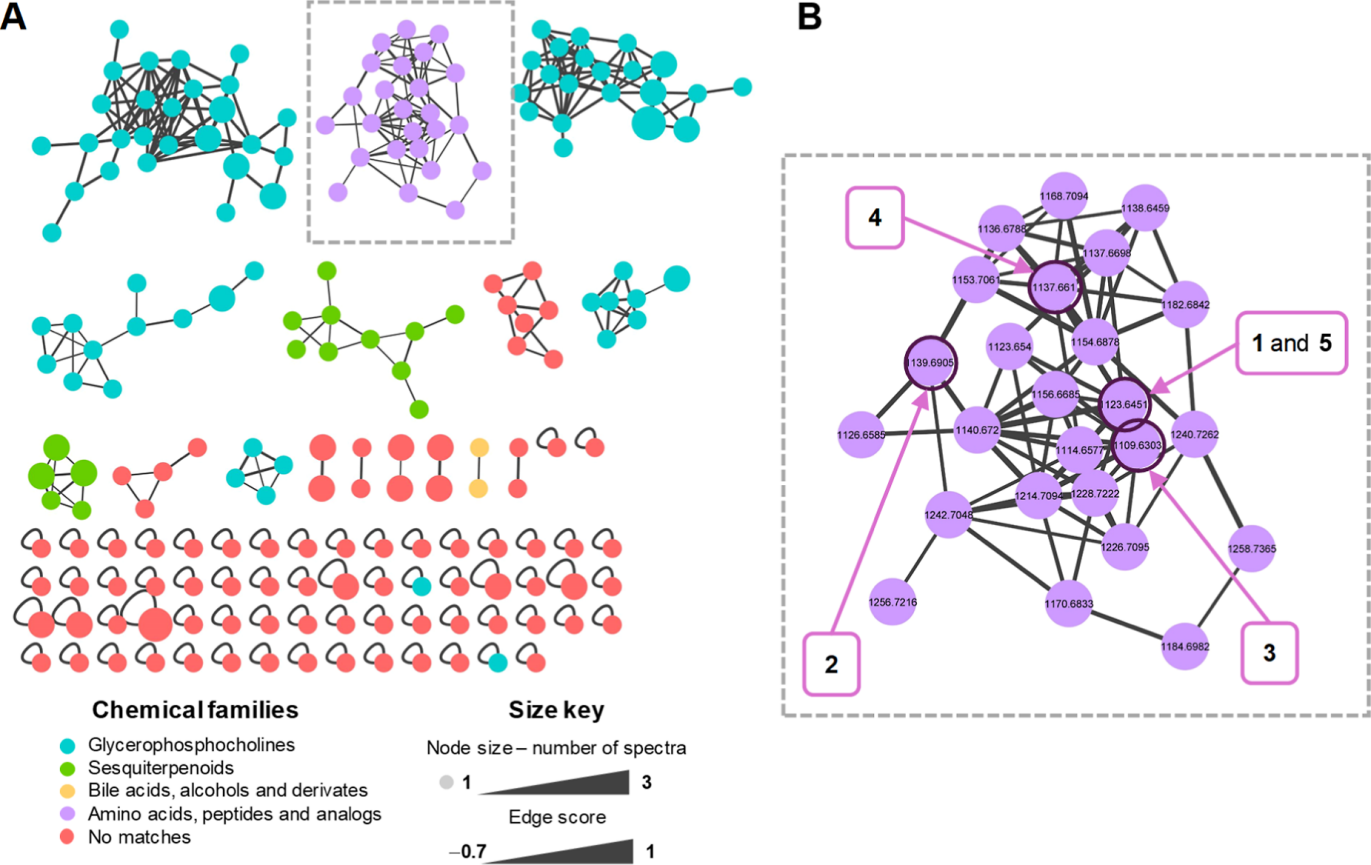
(A) Metabolomic analysis of the CR34 organic extract. (B) Peptide cluster with nodes showing the compounds annotated (in purple circles).

**Figure 3:**
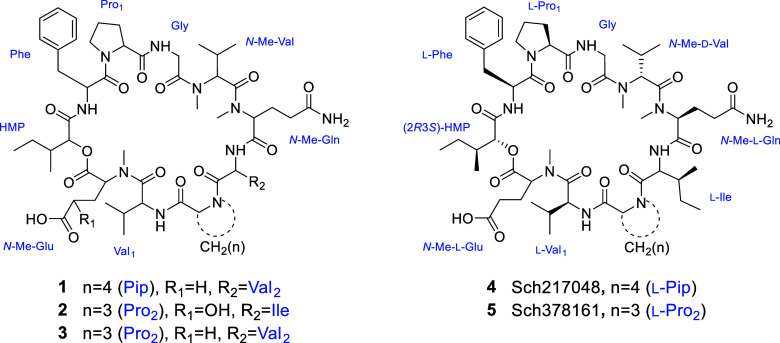
Isolated cyclodepsipeptides from fungus CR34.

During our search for new specialized metabolites with antimicrobial activity from unexplored sources, we isolated an unidentified aquatic fungus (CR34) from decaying wood samples submerged at the waterfall “El Caracol” in Iturbide, Nuevo Leon, at the SMO (Figure 1), which showed modest antimicrobial activity against *Acinetobacter baumannii* ATCC 17978 and a multidrug-resistant clinical strain A564 (∼50% inhibition; data not shown). The defatted organic extract of the fungus was also subjected to untargeted metabolomic analysis via feature-based molecular networking (FBMN) using high-resolution mass spectrometry (HRMS)–MS/MS data (Figure 2).^14−16^ The MN’s metabolite features were grouped into 197 nodes arranged in 10 clusters with >3 nodes per cluster, six with two nodes, and 64 singletons. Chemical ontology analysis classified the molecular features into ten classes of compounds, with the second-largest family in the network belonging to amino acids, peptides, and analogues (MW between 1109 and 1258 Da). Finally, using a bioactive-guided protocol, we were able to isolate three undescribed cyclodepsipeptides (**1**–**3**) and the known Sch217048 (**4**) and Sch378161 (**5**) (Figure 3).^17,18^ This class of peptides is constituted by ten amino acids and is produced by both bacteria and fungi (mainly ascomycetes). Only a few of such peptides contain a pipecolic acid (Pip) residue in their core.^19^ Moreover, compounds **4** and **5** and analogues Sch378199 and Sc378167 showed selective inhibitory activity on the neurokinin tachykinin receptors (NK_2_) implicated in edema and neurogenic inflammation,^17−21^ with no antimicrobial or cytotoxic activity.^21,22^

## Results and Discussion

Chromatographic fractionation of the defatted extract of fungus CR34 by flash chromatography on Si-gel and purification of an active primary fraction by preparative high-performance liquid chromatography (HPLC) on C_18_ (see Experimental Section for details) yielded cyclodepsipeptides Sch 217048 (**4**) and Sch 378161 (**5**), along with three new analogues (**1**–**3**). The structure of **4** and **5** was established by comparison of the one-dimensional (1D) and two-dimensional (2D) nuclear magnetic resonance (NMR) data and HRESIMS and MS/MS fragmentation data with those reported in the literature (Figures S25–S28 and Table S1).^17,18,20−22^ The amino acid sequence of **4** and **5** is practically the same, except that **4** contains a Pip residue instead of a proline (Pro) found in **5**. This particular difference was crucial to elucidating the structures of compounds **1**–**3**.

**Figure 4:**
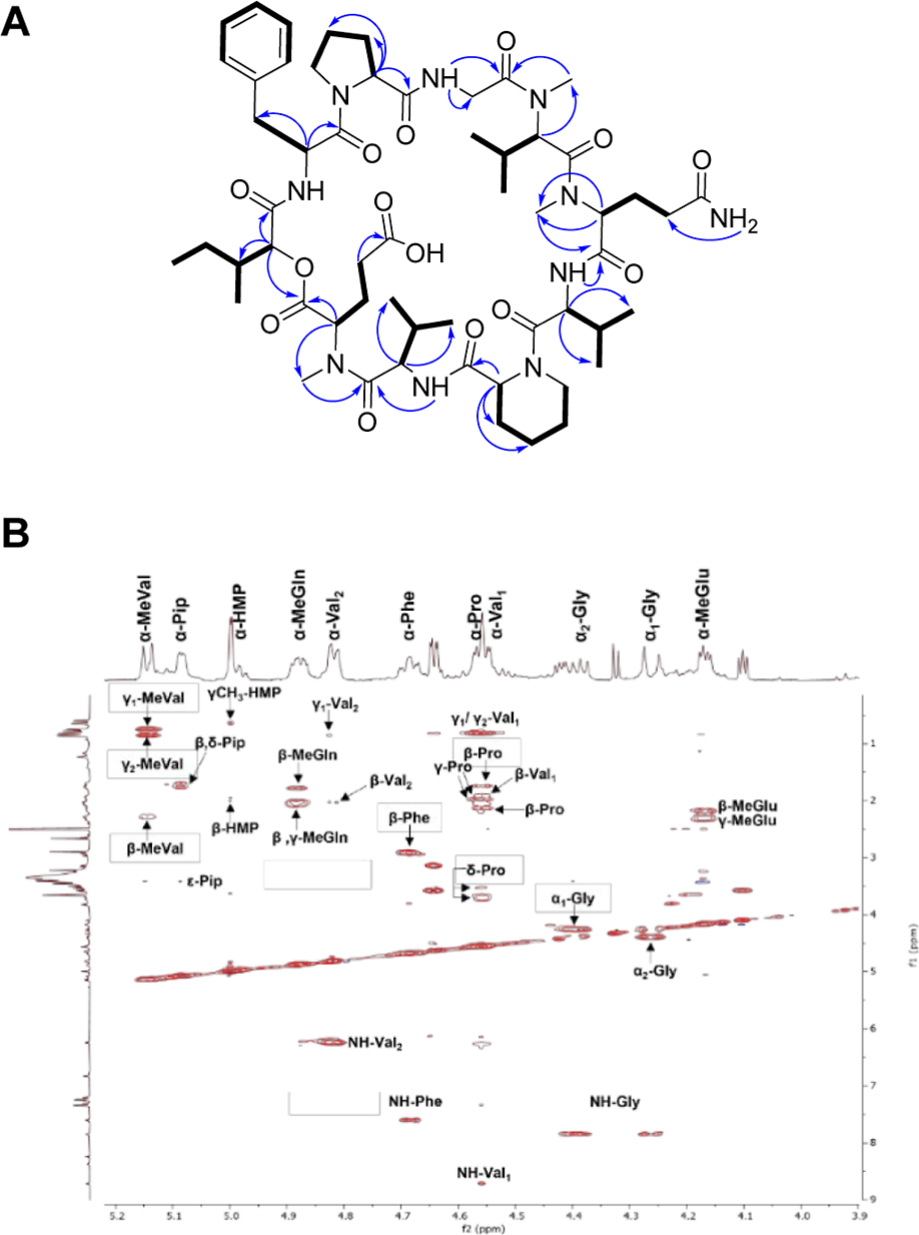
(A) Key COSY (bold lines) and HMBC (→) correlations observed in **1**. (B) Expansion of the TOCSY spectrum of **1** (f1 = 0.0–9.0 ppm, f2 = 3.9–5.2 ppm).

**Figure 5:**
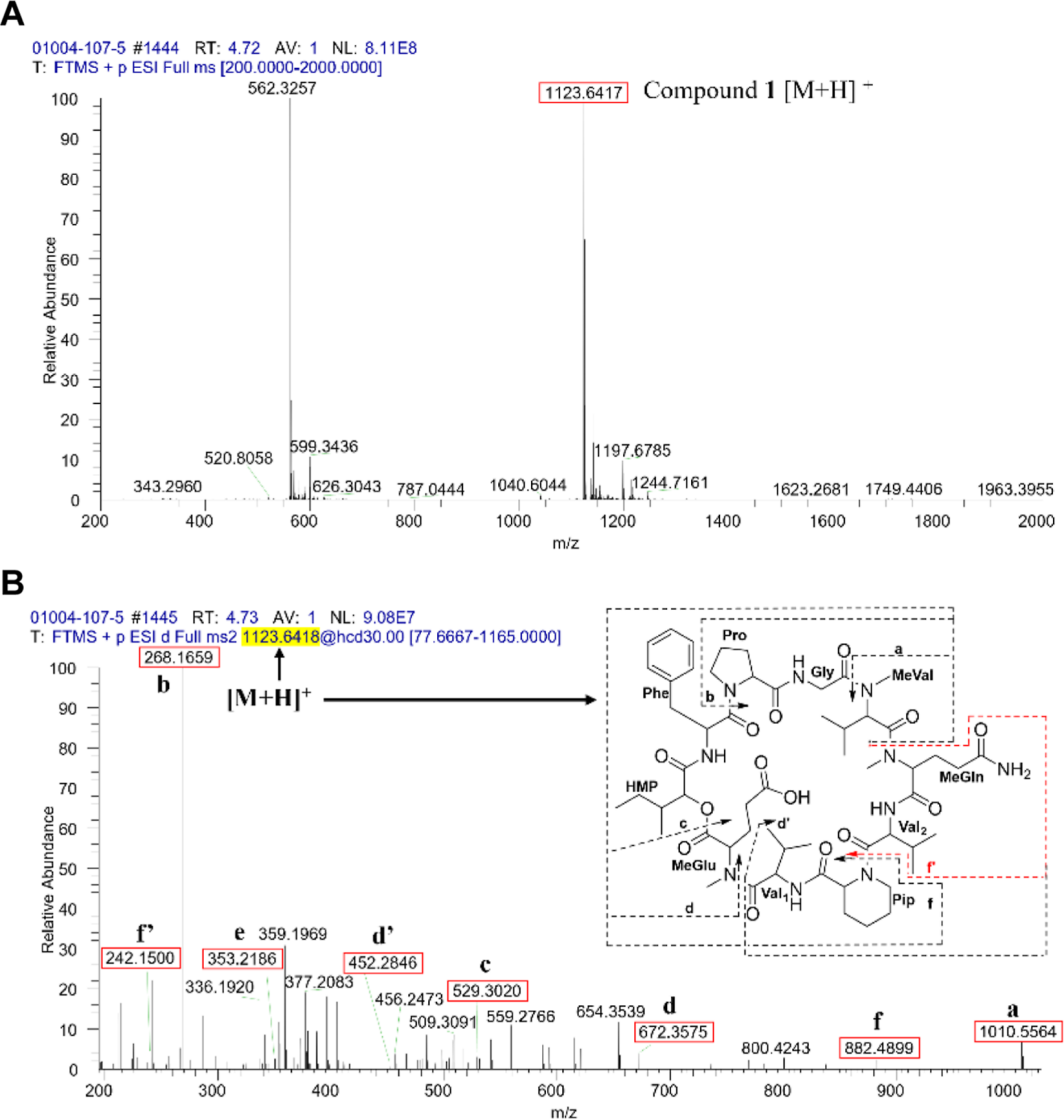
(A) Full-scan HRESIMS spectra of **1** and (B) positive HRESIMS–MS/MS mass fragmentation patterns showing the amino acid losses from the molecular ion *m*/*z* 1123.6418 [M + H]^+^.

Compound **1** was obtained as a white amorphous solid, and its molecular formula was determined as C_56_H_86_N_10_O_14_ based on the HRESIMS ion peak at *m*/*z* 1123.6417 [M + H]^+^ (calcd. C_56_H_87_N_10_O_14_, Δ = +1.7 ppm; IDH = 19). The analysis of the NMR and HRESIMS data of **1** (Tables 1 and 2 and Figures 4, 5, and S1–S8) showed structural similarity to **4**. The key −14 Da difference and the missing methylene at δ_H_ 1.26 (m) ppm in **1** suggested that this compound contains a valine (Val) instead of an isoleucine (Ile) as in **4**. This was set by the presence of a methine in **1** at δ_H_ 2.02 (1H, m, β-Val_2_) and δ_C_ 29.2 (β-Val_2_) and its COSY correlations with both methyl groups (γ_1_-Val_2_ and γ_2_-Val_2_) (Figure 4). In addition, the ^13^C and HSQC spectra (Figures S2 and S3) confirmed the presence of 56 carbons: 12 carbonyls, 6 aromatics (5 protonated), 13 methylenes, 14 methines, and 11 methyls (3 *N*–Me). Also, the HMBC and TOCSY data (Figures 4, S4, and S5) and the MS/MS fragmentation analysis were particularly helpful in establishing the amino acid sequence and identifying each spin system in **1**. The connection of Pip-Val_2_-*N*-MeGln was confirmed by the HMBC correlations between the NH (δ_H_ 6.25) of Val_2_ to the carbonyl (δ_C_ 168.6) of *N*-MeGln and the α-H (δ_H_ 5.08) of Pip to the carbonyl (δ_C_ 170.1) of Val_2_. Finally, the MS/MS analysis (Figure 5 and Table 3) revealed fragmentation patterns generated from ions containing the Pip residue at *m*/*z* 1010.5564 [*N*-MeGln-Val_2_-Pip-Val_1_-*N*-MeGlu-HMP-Phe-Pro_1_-Gly + H]^+^, *m*/*z* 452.2846 [*N*-MeGln-Val_2_-Pip-Val_1_ + H]^+^, *m*/*z* 353.2186 [*N*-MeGln-Val_2_-Pip + H]^+^, and *m*/*z* 882.4899 [*N*-MeVal-Gly-Pro_1_-Phe-HMP-*N*-MeGlu-Val_1_-Pip + H]^+^. The planar structure of **1** was thus confirmed as cyclo-(*N*-MeVal-*N*-MeGln-Val_2_-Pip-Val_1_-*N*-MeGlu-HMP-Phe-Pro-Gly).

**Table 1:** ^1^H NMR Data for **1**–**3** in DMSO-*d*_6_ at 700 MHz [δ_H_, mult (*J* in Hz) in ppm]

amino acid		1	2	3	amino acid		1	2	3
*N*-MeGlu/*N*-MeGlu(γ–OH)	α	4.16. dd (8.0, 4.0)	4.19, dd (8.0, 3.3)	4.23, m	*N*-MeVal	α	5.14, d (9.0)	5.05, d (10.4)	5.06, d (10.1)
	β	2.28, m	2.30, m	2.27, m		β	2.88, m	2.27, m	2.27, m
	γ	2.33, m	4.22, m	1.95, m		γ_1_-Me	0.74, d (6.7)	0.72, d (6.7)	0.72, d (6.4)
	NMe	3.22, s	3.25, s	3.23, s		γ_2_-Me	0.85, m	0.85, m	0.85, d (6.4)
Val_1_	α	4.81, dd (8.5, 2.5)	4.65, m	4.61, t (9.5)		NMe	2.90, s	2.89, s	2.90, s
	β	2.02, m	1.94, m	1.95, m	Gly	α	4.40, dd (15.5, 7.7)	4.46, m	4.44 dd (17.6, 8.8)
							4.26, d (15.5)	4.15, d (17.0)	4.27, d (16.4)
	γ_1_-Me	0.81, m	0.84, d (6.7)	0.76, d (6.4)		NH	7.84, d (7.5)	7.76, d (9.0)	7.93, d (9.0)
	γ_2_-Me	0.59, d (6.7)	0.92, d (6.7)	0.88, d (6.4)	Pro_1_	α	4.56, m	4.40, dd (7.5, 5.2)	4.52, dd (8.5, 3.6)
	NH	8.71, d (8.4)	8.47, d (9.0)	8.48, d (10.1)		β	2.13, m	2.14, m	2.15, m
							1.74, m	1.77 m	1.75, m
Pip/Pro_2_	α	5.08, dd (5.0, 4.5)	4.65, m	4.46, dd (8.1, 5.7)		γ	2.05, m	2.10, m	1.95, m
							1.92, m	1.92, m	2.08, m
	β	1.76 m	2.18, m	1.95, m		δ	3.72, m	3.74, m	3.68, m
		1.71, m	1.74, m	1.55, m			3.53, m	3.58, m	3.41, m
	γ	1.42, m	2.08, m	2.03, m	Phe	α	4.68, dd (8.0, 3.0)	4.70, dt (9.2, 5.1)	4.66, dd (8.0, 5.0)
			1.87 m	1.83, m					
	δ	1.70, m	3.70, m	3.71, m		β	2.92, m	2.95, m	2.93, m
			3.50, m	3.46, m					
	ε	3.82, m				γ-C_2/_C_6_	7.34, m	7.33, m	7.32, m
		3.69, m							
Val_2_/Ile	α	4.56, dt (8.0, 4.0)	4.57, dd (8.0, 4.0)	4.57, m		γ-C_3/_C_5_	7.24, m	7.24, m	7.24, m
	β	1.97, m	1.73, m	1.95, m		γ-C_4_	7.20, m	7.20, m	7.20, m
	γ_1_-Me	0.81, m	0.85, d (6.7)	0.92, d (6.4)		NH	7.60, d (8.0)	7.68, d (8.3)	7.80, d (9.0)
	γ-CH_2_		1.40, m		HMP	α	4.99, bd (1.7)	4.88, d (2.2)	4.91 d (2.1)
	γ_2_-Me	0.86, m	0.80, d (6.7)	0.83, d (6.4)		β	1.94, m	1.93, m	1.95, m
	NH	6.25, d (8.0)	6.29, d (8.0)	6.21 (8.4)		γ-Me	0.64, d (6.7)	0.66, d (6.7)	0.66 d (6.4)
*N*-MeGln	α	4.88, dd (8.0, 3.0)	4.81, m	4.81, dd (8.6, 3.0)		γ-CH_2_	1.17, m	1.18, m	1.17, m
	β	2.18, m	2.12 m	2.08, m		δ-Me	0.77, d (6.7)	0.78, d (6.7)	0.77 d (6.4)
		1.77, m	1.75, m	1.75, m					
	γ	2.08, m	2.02, m	2.08, m					
		2.01, m	1.97, m	1.95, m					
	δ-CONH_2_	7.31, bs	7.34, bs	7.34, bs					
		6.83, bs	6.80, bs	6.81, bs					
	NMe	2.66, s	2.64, s	2.65, s					

**Table 2:** ^13^C NMR Data for **1**–**3** in DMSO-*d*_6_ at 175 MHz [δ_C_ in ppm]

amino acid		1	2	3	amino acid		1	2	3
*N*-MeGlu/*N*-MeGlu(γ–OH)	CO	169.6	169.6	168.2	*N*-MeVal	CO	169.8	170.9	170.0
	α	62.0	62.4	62.5		α	57.1	57.2	57.2
	β	23.9	25.0	24.1		β	27.5	27.0	27.1
	γ	30.6	69.3	31.1		γ_1_-Me	18.0	18.0	18.0
	δ-COOH	174.2	173.6	173.5		γ_2_-Me	19.2	19.4	19.4
	NMe	38.5	38.9	38.8		NMe	28.2	28.4	28.3
Val_1_	CO	170.1	171.4	169.9	Gly	CO	170.6	170.0	170.4
	α	52.5	53.7	53.8		α	41.3	40.3	40.5
	β	29.2	31.9	31.7	Pro_1_	CO	170.7	170.1	170.9
	γ_1_-Me	19.2	19.1	17.0		α	59.5	59.8	59.4
	γ_2_-Me	15.5	18.0	19.1		β	29.1	28.9	28.9
Pip/Pro_2_	CO	172.2	169.9	171.7		γ	24.9	25.3	25.0
	α	52.5	58.1	59.6		δ	47.1	47.1	47.1
	β	27.1	28.9	29.9	Phe	CO	169.8	168.3	168.4
	γ	19.7	24.6	24.5		α	52.3	52.2	52.3
	δ	24.5	48.6	47.6		β	36.4	36.4	36.3
	ε	43.1				γ-C_1_	137.5	137.5	137.7
Val_2_/Ile	CO	172.4	172.4	172.3		C_2/_C_6_	129.4	129.4	129.4
	α	54.2	54.0	54.2		C_3/_C_5_	128.2	128.2	128.2
	β	31.1	37.5	31.1		C_4_	126.5	126.5	126.4
	γ_1_-Me	17.9	14.9	18.0	HMP	CO	168.1	168.2	166.4
	γ_2_-Me	19.2	11.1	19.2		α	74.6	75.0	74.7
	γ-CH_2_		23.7			β	35.7	36.0	35.9
*N*-MeGln	CO	168.7	168.9	168.1		γ-Me	13.9	14.6	14.6
	α	58.9	59.0	59.0		γ-CH_2_	25.4	25.5	25.5
	β	24.1	24.1	24.1		δ-Me	11.6	11.6	11.6
	γ	30.9	31.0	31.0					
	δ-CONH_2_	173.3	173.2	173.2					
	NMe	29.3	29.4	29.4					

**Table 3:** HRESIMS–MS/MS Mass Fragmentation Patterns of **1** Showing the Amino Acid Losses from the Molecular Ion [M + H]^+^

	fragment annotation^a^	measured *m*/*z*	calculated *m*/*z*	molecular formula	mass accuracy (ppm)	IDH
a	[*N*-MeGln-Val_2_-Pip-Val_1_-*N*-MeGlu-HMP-Phe-Pro_1_-Gly + H]^+^	1010.5564	1010.5562	C_50_H_76_N_9_O_13_	0.7	18
b	[*N*-MeVal-Gly-Pro_1_ + H]^+^	268.1659	268.1661	C_13_H_22_N_3_O_3_	1.2	5
c	[*N*-MeVal-Gly-Pro_1_-Phe-HMP + H]^+^	529.3020	529.3025	C_28_H_41_N_4_O_6_	–0.1	11
d	[*N*-MeVal-Gly-Pro_1_-Phe-HMP-*N*-MeGlu + H]^+^	672.3575	672.3608	C_34_H_50_N_5_O_9_	–4.2	13
d’	[*N*-MeGln-Val_2_-Pip-Val_1_ + H]^+^	452.2846	452.2872	C_22_H_38_N_5_O_5_	–4.7	7
e	[*N*-MeGln-Val_2_-Pip + H]^+^	353.2186	353.2188	C_17_H_29_N_4_O_4_	0.8	6
f	[*N*-MeVal-Gly-Pro_1_-Phe-HMP-*N*-MeGlu-Val_1_-Pip + H]^+^	882.4899	882.4976	C_45_H_68_N_7_O_11_	–8.2	16
f^b^	[*N*-MeGln-Val_2_ + H]^+^	242.1500	242.1504	C_11_H_20_N_3_O_3_	0.3	4

a The amide bond *N*-MeVal-*N*-MeGln corresponds to the fragmentation starting point (clockwise and counterclockwise).

b Key fragment indicates the presence of Val_2_ bonded to *N*-MeGln instead of Ile.

**Figure 6:**
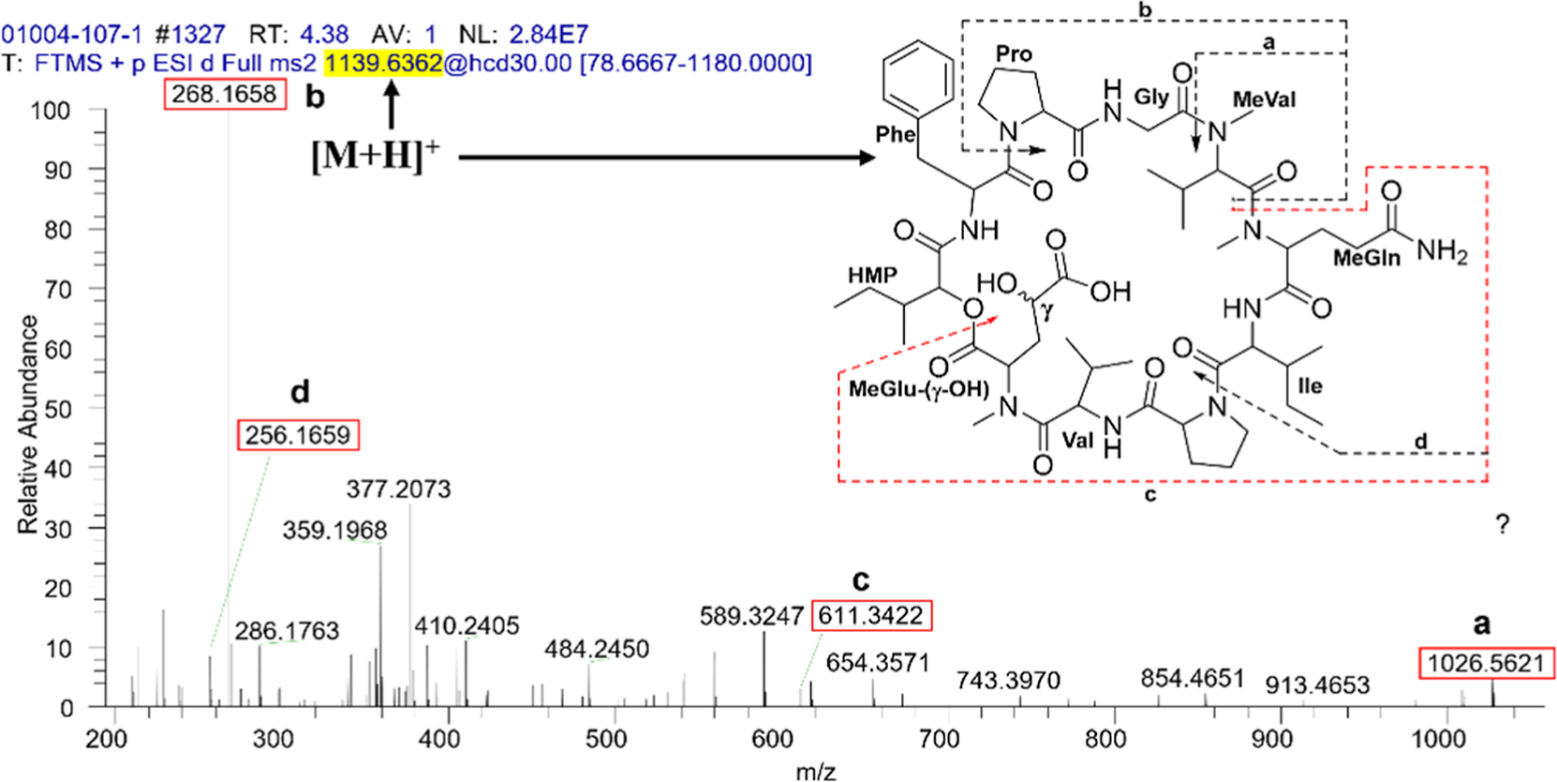
HRESIMS–MS/MS mass fragmentation patterns spectra of **2** showing the amino acid losses from the molecular ion *m*/*z* 1139.6362 [M + H]^+^.

Compound **2** was isolated as a white, amorphous solid. Its molecular formula was deduced as C_56_H_86_N_10_O_15_ based on the HRESIMS ion peak at *m*/*z* 1139.6362 [M + H]^+^ (calcd. C_56_H_87_N_10_O_15_, Δ = +1.3 ppm, IDH = 19) (Figure S16). Detailed analysis of the 1D and 2D NMR data (Tables 1 and 2 and Figures S9–S15) showed that this compound also has a cyclodepsipeptide core with an NMR profile like Sch 378161 (**5**). The main difference between these compounds is the presence of hydroxymethine at the γ-position of *N*-MeGlu in **2** (δ_H_ 4.22, m; δc 69.3 ppm), instead of the characteristic γ-methylene in **5** (δ_H_ 2.33, m; δ_C_ 31.0 ppm), which was confirmed by the HSQC and HMBC correlations (Figures S11 and S12**)**. In addition, 16 Da more compared to **5** observed in the HRESIMS of **2**, and its MS/MS fragmentation pattern (Figure 6 and Table 4**)** confirmed the addition of an O atom at the side chain of the *N*-MeGlu residue. The γ-oxidation of the Glu residue is possible due to the formation of the γ-carbon peroxyl radical and subsequent reactions leading to the formation of side chain modification products.^23^ Thus, the amino acid sequence and planar structure of **2** were then deduced as cyclo-(*N*-MeVal-*N*-MeGln-Ile-Pro_2_-Val_1_-*N*-MeGlu(γ–OH)-HMP-Phe-Pro_1_-Gly).

**Table 4:** HRESIMS–MS/MS Mass Fragmentation Patterns of **2** Showing the Amino Acid Losses from the Molecular Ion [M + H]^+^

	fragment annotation^a^	measured *m*/*z*	calculated *m*/*z*	molecular formula	mass accuracy (ppm)	IDH
a	[*N*-MeGln-Ile-Pro_2_-Val_1_-*N*-MeGlu-(γ–OH)-HMP-Phe-Pro_1_-Gly + H]^+^	1026.5621	1026.5511	C_50_H_76_N_9_O_14_	0.5	18
b	[*N*-MeVal-Gly-Pro_1_ + H]^+^	268.1658	268.1661	C_13_H_22_N_3_O_3_	0.9	5
c^b^	[*N*-MeGln-Ile-Pro_2_-Val-*N*-MeGln-(γ–OH) + H]^+^	611.3422	611.3326	C_28_H_47_N_6_O_9_	3.8	9
d	[*N*-MeGln-Ile + H]^+^	256.1659	256.1658	C_12_H_22_N_3_O_3_	0.9	4

a The amide bond *N*-MeVal-*N*-MeGln corresponds to the fragmentation starting point (clockwise and counterclockwise).

b Key fragment indicates the presence of a hydroxymethine group in the amino acid *N*-MeGlu.

**Figure 7:**
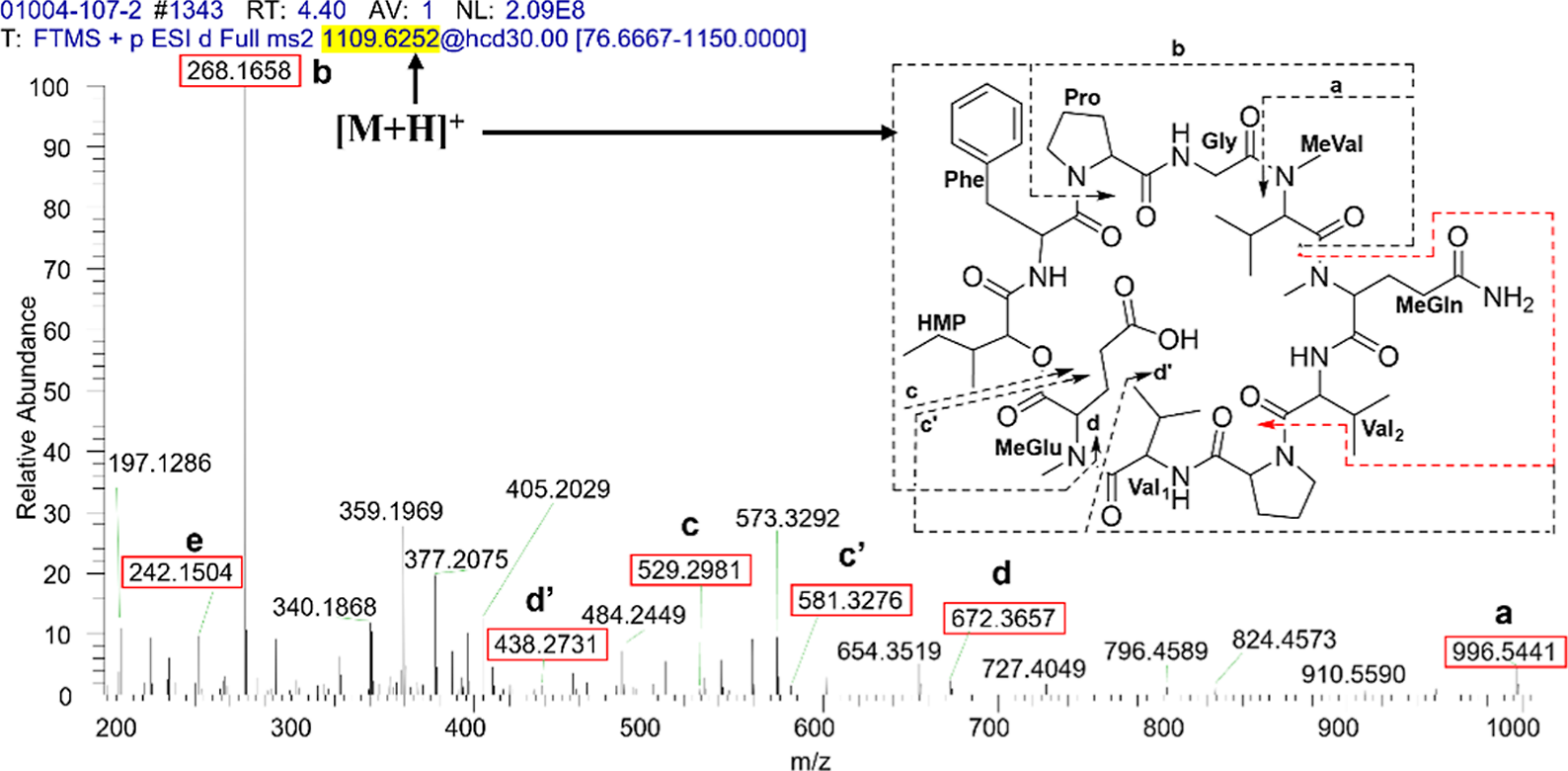
HRESIMS–MS/MS mass fragmentation patterns spectra of **3** showing the amino acid losses from the molecular ion *m*/*z* 1109.6252 [M + H]^+^.

Compound **3** was isolated as a white amorphous solid, and its molecular formula was determined as C_55_H_84_N_10_O_14_ based on the HRESIMS ion peak at *m*/*z* 1109.6257 [M + H]^+^ (calcd. C_55_H_85_N_10_O_14_, Δ = +1.4 ppm, IDH = 19) (Figure S24). The ^1^H and ^13^C NMR data (Tables 1 and 2 and Figures S17–S23) confirmed its cyclodepsipeptide core, and the difference of −14 Da compared to that of **5** indicated the loss of a methylene group. This was also established by the presence of a β-methine, characteristic of a Val_2_ residue at δ_H_ 1.95 (1H, m) and δ_C_ 31.1, instead of the typical resonances of an Ile in **5** (Tables 1 and 2). The MS/MS analysis (Figure 7 and Table 5) revealed the fragmentation pattern that allowed us to establish the amino acid sequence of **3** as cyclo-(*N*-MeVal-*N*-MeGln-Val_2_-Pro_2_-Val_1_-*N*-MeGlu-HMP-Phe-Pro_1_-Gly).

**Table 5:** HRESIMS–MS/MS Mass Fragmentation Patterns of **3** Showing the Amino Acid Losses from the Molecular Ion [M + H]^+^

fragment annotation^a^		measured *m*/*z*	calculated *m*/*z*	molecular formula	mass accuracy (ppm)	IDH
a	[*N*-MeGln-Val_2_-Pro_2_-Val_1_-*N*-MeGlu-HMP-Phe-Pro_1_-Gly + H]^+^	996.5441	996.5406	C_49_H_74_N_9_O_13_	4.1	18
b	[*N*-MeVal-Gly-Pro_1_ + H]^+^	268.1658	268.1661	C_13_H_22_N_3_O_3_	0.9	5
c	[*N*-MeVal-Gly-Pro_1_-Phe-HMP + H]^+^	529.2981	529.3026	C_28_H_41_N_4_O_6_	–7.5	11
c′	[*N*-MeGln-Val_2_-Pro_2_-Val_2_-*N*-MeGlu + H]^+^	581.3276	581.3298	C_27_H_45_N_6_O_8_	–3.0	9
d	[*N*-MeVal-Gly-Pro_1_-Phe-HMP-*N*-MeGlu + H]^+^	672.3657	672.3608	C_34_H_50_N_5_O_9_	8.0	13
d′	[*N*-MeGln-Val_2_-Pro_2_-Val_1_ + H]^+^	438.2731	438.2716	C_21_H_36_N_5_O_5_	4.6	7
e^b^	[*N*-MeGln-Val_2_ + H]^+^	242.1504	242.1504	C_11_H_20_N_3_O_3_	2.0	4

a The amide bond *N*-MeVal-*N*-MeGln corresponds to the fragmentation starting point (clockwise and counterclockwise).

b Key fragment: indicates the presence of Val_2_ bonded to *N*-MeGln instead of Ile.

Unfortunately, due to the scarcity of samples isolated, the absolute configuration of the amino acid sequence of **1**–**3** was not established. Also, due to the latter, only compounds **4** and **5** were evaluated for their antimicrobial activity against a panel of 12 human pathogens (Table 6).^24−26^ These compounds showed moderate activity against vancomycin-susceptible and -resistant *Enterococcus faecalis*, methicillin-susceptible and -resistant *Staphylococcus aureus*, and against a sensible and a clinically isolated multidrug-resistant *A. baumannii* at the concentrations tested, 0.09 μM (100 ppm) and 0.01 μM (10 ppm). Interestingly, while the inhibition of *A. baumannii* was under 40%, this information represents an important contribution of peptides with ≤10 amino acids for antimicrobial drug discovery against this pathogen.^27,28^ Finally, **4** and **5** did not show toxicity in the *Galleria mellonella* larvae model (Figure S29).^26,29−32^

**Table 6:** Antimicrobial Activity of **4** and **5**

	% growth inhibition											
	VSEF^a^		VREF^b^		MSSA^c^		MRSA^d^		*A. baumannii*^e^		*A. baumannii*^f^	
compound	0.09 μM	0.01 μM	0.09 μM	0.01 μM	0.09 μM	0.01 μM	0.09 μM	0.01 μM	0.09 μM	0.01 μM	0.09 μM	0.01 μM
Sch 217048 (**4**)	20.1	14.5	37.5	–5.8	43.8	17.2	41.8	15.0	13.3	–1.1	35.5	3.6
Sch 378161 (**5**)	24.2	3.5	18.9	12.1	46.6	17.7	42.9	13.6	–6.7	–3.0	–5.0	3.6
MIC positive control (μg/mL)	3.8^g^	162.5^g^	0.1^h^	2.5^g^	5.0^h^	6400.0^i^						

a VSEF: vancomycin-susceptible *E. faecalis* ATCC 29212.

b VREF: vancomycin-resistant *E. faecalis* ATCC 51299.

c MSSA: methicillin-susceptible *S. aureus* ATCC 25923.

d MRSA: methicillin-resistant *S. aureus* ATCC 43300.

e *A. baumannii* gentamicin-susceptible ATCC 17978.

f Clinical isolated *A. baumannii* multidrug-resistant A564.

g Vancomycin.

h Ampicillin.

i Gentamicin.

In summary, this work represents the first chemical study of a fungal strain isolated from submerged wood in a waterfall at the Sierra Madre Oriental (SMO) in Mexico. From the extract of rice culture, five cyclodepsipeptides were isolated, including known Sch 217048 (**4**) and Sch 378161 (**5**). Both **4** and **5** showed antimicrobial activity on Gram-positive strains and the Gram-negative *A. baumannii*, including a clinical multidrug-resistant strain. The compounds showed no toxic activity in the *G. mellonella* larvae model. Overall, this work provides interesting insight into the activity of peptides for antimicrobial drug discovery against this pathogen.

## Experimental Section

### General Experimental Procedures

NMR experiments were conducted in dimethyl sulfoxide (DMSO-*d*_6_) using either a Varian Inova 700 NMR spectrometer (Varian Inc., Palo Alto, CA) equipped with a high-sensitivity Varian cold probe or a JEOL ECA-500 (JEOL Ltd., Japan) spectrometer equipped with a high-sensitivity JEOL Royal probe. LC-HRESIMS data were collected using an Acquity UPLC system (Waters, Milford, MA) coupled to a Q Exactive Plus system (Thermo Fisher Scientific, Waltham, MA) equipped with an electrospray ionization source (positive and negative ionization modes) with an HCD cell and a BEH C_18_ column (50 × 2.1 mm i.d., 1.7 μm, 130 Å; Waters) with a gradient solvent system from 15:85 to 100:0 CH_3_CN–H_2_O (both phases acidulated with 0.1% formic acid) for 10 min at a flow rate of 0.3 mL/min. Analytical and preparative HPLC were performed on a Waters HPLC system equipped with a 2535 quaternary pump, a 2707 autosampler, a 2998 potato dextrose agar (PDA) cell, a 2424 ELSD cell, and a fraction collector III (only for preparative mode) using a Gemini C_18_ column (analytical: 250 × 4.6 mm i.d., 5 μm, 100 Å; preparative: 250 × 21.2 mm i.d., 5 μm, 100 Å; Phenomenex, Torrance, CA) using different gradient solvent systems. Flash chromatography was performed on a CombiFlash Rf^+^ Lumen system (Teledyne Technologies Inc., Thousand Oaks, CA) equipped with PDA and ELSD detectors and using RediSep *R*_f_ Gold-Sigel columns (20–40 μm spherical, 60 Å, Teledyne Technologies Inc.). Reagents, ACS, HPLC, and MS grade, were purchased from J.T. Baker (Avantor Performance Materials, Center Valley, PA).

### Fungal Strain Isolation and Identification

The fungal strain CR34 was isolated from submerged dead and decaying wood samples collected at the waterfall “El Caracol” (24°46′58.6″N 99°54′31.3″W, downstream), in Iturbide, Nuevo Leon, Mexico, during March 2021. Briefly, after collecting the submerged wood, the materials were incubated at room temperature with sterile, moist paper towels for four months in 12 h light/dark cycles until the fruiting bodies were observed. Then, the ascomata were spread on antibiotic water agar plates with 0.5 g of streptomycin/L and 0.5 g of ampicillin/L, and the germinating mycelium was transferred to PDA. After 5 days, agar plugs were transferred to yeast extract–soy peptone–dextrose media and incubated for 7 days at room temperature and then transferred to five Erlenmeyer flasks with rice media (15 g and 30 mL of H_2_O) for 21 days at room temperature to obtain the final extract. Unfortunately, after several attempts at DNA extraction and recultivation of the strain, it was impossible to obtain the ITS sequencing information required for identification. Interestingly, this is not the first example of a cyclodepsipeptide-fungal producer that has not been identified.^17,18,20^

### Fermentation, Extraction, and Isolation

The extract from the rice-media cultures was obtained by maceration with CHCl_3_–CH_3_OH and then defatted using *n*-hexane. The final extract (834.3 mg) was adsorbed on Celite 545 (Thermo Fisher Scientific) and fractionated via flash chromatography on a 24 g RediSep Rf Gold Si-gel column using a gradient solvent system of *n*-hexane-CHCl_3_–CH_3_OH at a flow rate of 35 mL/min. For the run, 50 column volumes were used, and fractions were collected every 15.0 mL and pooled according to the UV and ELSD profiles. From the 12 primary fractions obtained, fraction 7 (111.8 mg) was subjected to preparative HPLC separation using a gradient from 30:70 to 80:20 CH_3_CN-0.1% aqueous formic acid in 15 min at 21.24 mL/min, yielding compounds **1** (3.0 mg, *t*_R_ = 12.9 min), **2** (1.1 mg, *t*_R_ = 11.0 min), **3** (1.7 mg, *t*_R_ = 11.7 min), **4** (20.8 mg, *t*_R_ = 13.7 min), and **5** (15.8 mg, *t*_R_ = 12.3 min).

#### Cyclo-(N-MeVal-N-MeGln-Val_2_-Pip-Val_1_-N-MeGlu-HMP-Phe-Pro_1_-Gly)-**1**

White amorphous solid (3.0 mg); UV λ_max_, 222 and 227 nm; NMR data, see Tables 1 and 2 and Supporting Information; HRESIMS *m*/*z* 1123.6417: [M + H]^+^ (calcd C_56_H_87_N_10_O_14_, 1123.6398 uma).

#### Cyclo-(N-MeVal-N-MeGln-Ile-Pro_2_-Val_1_-N-MeGlu(γ–OH)-HMP-Phe-Pro_1_-Gly)-**2**

White amorphous solid (1.1 mg); UV λ_max_, 222 and 227 nm; NMR data, see Tables 1 and 2 and Supporting Information; HRESIMS *m*/*z* 1139.6365: [M + H]^+^ (calcd C_56_H_87_N_10_O_15_, 1139.6347 uma).

#### Cyclo-(N-MeVal-N-MeGln-Val_2_-Pro_2_-Val_1_-N-MeGlu-HMP-Phe-Pro_1_-Gly)-**3**

White amorphous solid (1.7 mg); UV λ_max_, 222 and 227 nm; NMR data, see Tables 1 and 2 and Supporting Information; HRESIMS *m*/*z* 1109.6251: [M + H]^+^ (calcd C_55_H_85_N_10_O_14_, 1109.6241 uma).

#### Sch 217048 (**4**)

White amorphous solid (20.8 mg); UV λ_max_, 222 and 227 nm; NMR data, see Table S1 and Supporting Information; HRESIMS *m*/*z* 1137.6576: [M + H]^+^ (calculated for C_57_H_89_N_10_O_14_, 1139.6554 uma).

#### Sch 378161 (**5**)

White amorphous solid (15.8 mg); UV λ_max_, 222 and 227 nm; NMR data, see Table S1 and Supporting Information; HRESIMS *m*/*z* 1123.6420: [M + H]^+^ (calculated for C_56_H_87_N_10_O_14_, 1123.6403 uma).

### Antimicrobial Assays

The extract, fractions, and pure compounds were evaluated in vitro for antibacterial activity using the Clinical and Laboratory Standards Institute reference broth dilution method.^24,25^ Target microorganisms used in the assays include bacteria from the ESKAPE group as well as an opportunistic yeast: vancomycin-susceptible *E. faecalis* ATCC 29219, vancomycin-resistant *E. faecalis* ATCC 51299, methicillin-susceptible *Staphylococcus aureus* ATCC 25923, methicillin-resistant *S. aureus* ATCC 43300, *Bacillus spizizenii* ATCC 6633, *A. baumannii* ATCC 17978, *A. baumannii* clinical isolated strain A564,^26^*Klebsiella aerogenes* ATCC 13048, *K. pneumoniae* ATCC 700603, *Enterobacter cloacae* 700323, *Pseudomonas aeruginosa* ATCC 27853, and *Candida albicans* ATCC 10231. The evaluated samples were dissolved in DMSO (final concentration 2%) to obtain a stock solution and then tested at a final concentration of 200 and 20 μg/mL (for extract and fractions) and 100 and 10 μg/mL (for pure compounds). The assays were carried out in 96-well plates in duplicate. The MTT reagent (5 mg/mL in DMSO) was used as a viability indicator, and the absorbance data were collected at 595 nm.

### In Vivo Assays in *G. mellonella*

Toxicity of vehicles (NaCl 0.9%; DMSO 2%; DMSO 10%; 20 μL of each solution) and compounds **4** and **5** (final concentration 100 μg/μL in DMSO 2% and DMSO 10%; 10 μL of compound +10 μL of NaCl 0.9%) were injected in *G. mellonella* larvae (*n* ≥ 3, size ≥3.5 cm, no melanization observed, two independent cultures per group). After this, injected worms were incubated at 37 °C and monitored daily for 5 days to finally determine the percentage of survival of each group tested according to previous reports.^26,29−32^

### Molecular Networking and Metabolomic Analysis

The extract was analyzed by LC–HRMS–MS/MS using previously described methodology.^14−16^ Raw data were converted to mxML format using the ProteoWizard tool MSConvert (version 3.022010-e15b3da), and the converted files were processed in MZMINE (version 3.3.0). Then, the FBMN^14^ was carried out by uploading the refined matrix on the Global Natural Products Social server. Molecular networks were generated by following the workflow previously described. Parameters: precursor ion mass tolerance 0.01 Da, MS/MS fragment ion tolerance 0.02 Da, minimum matched peak 4, and cosine score 0.7. Molecular networks were visualized with Cytoscape 3.8.1.^33^ Finally, manual annotation of isolated compounds was at confidence level 1, according to the metabolomics standards initiative and exact mass accuracy <5 ppm.^34^

## Supporting Information Available

NMR and MS spectra and data of **1**–**5** and toxicity test results in *G. mellonella* larvae of **4** and **5** (PDF)
