# Supplementary material for: New Antimicrobial Cyclodepsipeptides from a Freshwater Fungus from the Sierra Madre Oriental in Mexico
Source: ACS Omega. 2025 Jan 29;10(5):5087–96. doi: 10.1021/acsomega.4c10990 (PMC11822689; doi:10.1021/acsomega.4c10990)
Supplement: Supplementary file 1 — ao4c10990_si_001.pdf [file ao4c10990_si_001.pdf]

Supporting Information

New antimicrobial cyclodepsipeptides from a freshwater fungus from the Sierra Madre Oriental in Mexico

Itzel Rubí Yeverino, Tania Paola Bocanegra Sosa, Laura Aguilar-Vega, Rodolfo García-Contreras, José L. Magaña-González, and Mario Figueroa*

*Corresponding Author: E-mail: mafiguer@unam.mx

Table of contents

Figure S1. ^{1}H NMR spectrum of **1** in DMSO-d_6 (700 MHz).
Figure S2. ^{13}C NMR spectrum of **1** in DMSO-d_6 (175 MHz).
Figure S3. HSQC spectrum of **1** in DMSO-d_6 (700 MHz).
Figure S4. HMBC spectrum of **1** in DMSO-d_6 (700 MHz).
Figure S5. TOCSY spectrum of **1** in DMSO-d_6 (700 MHz).
Figure S6. COSY spectrum of **1** in DMSO-d_6 (700 MHz).
Figure S7. NOESY spectrum of **1** in DMSO-d_6 (700 MHz).
Figure S8. UPLC-PDA (λ 227 nm) spectra of **1**.
Figure S9. ^{1}H NMR spectrum of **2** in DMSO-d_6 (700 MHz).
Figure S10. ^{13}C NMR spectrum of **2** in DMSO-d_6 (175 MHz).
Figure S11. HSQC spectrum of **2** in DMSO-d_6 (700 MHz). (A) Full spectrum; (B) Comparison of region of addition CH in *N*-MeGlu of **2** (left) and **5** (right).
Figure S12. HMBC spectrum of **2** in DMSO-d_6 (700 MHz). (A) Full spectrum; (B) Key correlations in *N*-MeGlu.
Figure S13. TOCSY spectrum of **2** in DMSO-d_6 (700 MHz).
Figure S14. COSY spectrum of **2** in DMSO-d_6 (700 MHz).
Figure S15. NOESY spectrum of **2** in DMSO-d_6 (700 MHz).
Figure S16. UPLC-PDA (λ 227 nm) and HRESIMS spectra of **2**.
Figure S17. ^{1}H NMR spectrum of **3** in DMSO-d_6 (700 MHz).
Figure S18. ^{13}C NMR spectrum of **3** in DMSO-d_6 (175 MHz).
Figure S19. HSQC spectrum of **3** in DMSO-d_6 (700 MHz).
Figure S20. HMBC spectrum of **3** in DMSO-d_6 (700 MHz).
Figure S21. TOCSY spectrum of **3** in DMSO-d_6 (700 MHz).
Figure S22. COSY spectrum of **3** in DMSO-d_6 (700 MHz).
Figure S23. NOESY spectrum of **3** in DMSO-d_6 (700 MHz).
Figure S24. UPLC-PDA (λ 227 nm) and HRESIMS spectra of **3**.
Figure S25. ^{1}H and ^{13}C NMR spectra of **4** in DMSO-d_6 (500 and 125 MHz, respectively).
Figure S26. UPLC-PDA (λ 227 nm) and HRESIMS spectra of **4**.
Figure S27. ^{1}H and ^{13}C NMR spectra of **5** in DMSO-d_6 (500 and 125 MHz, respectively).
Figure S28. UPLC-PDA (λ 227 nm) and HRESIMS spectra of **5**.
Figure S29. Toxicity results for the vehicles and **4** and **5** on *G. mellonella* larvae.

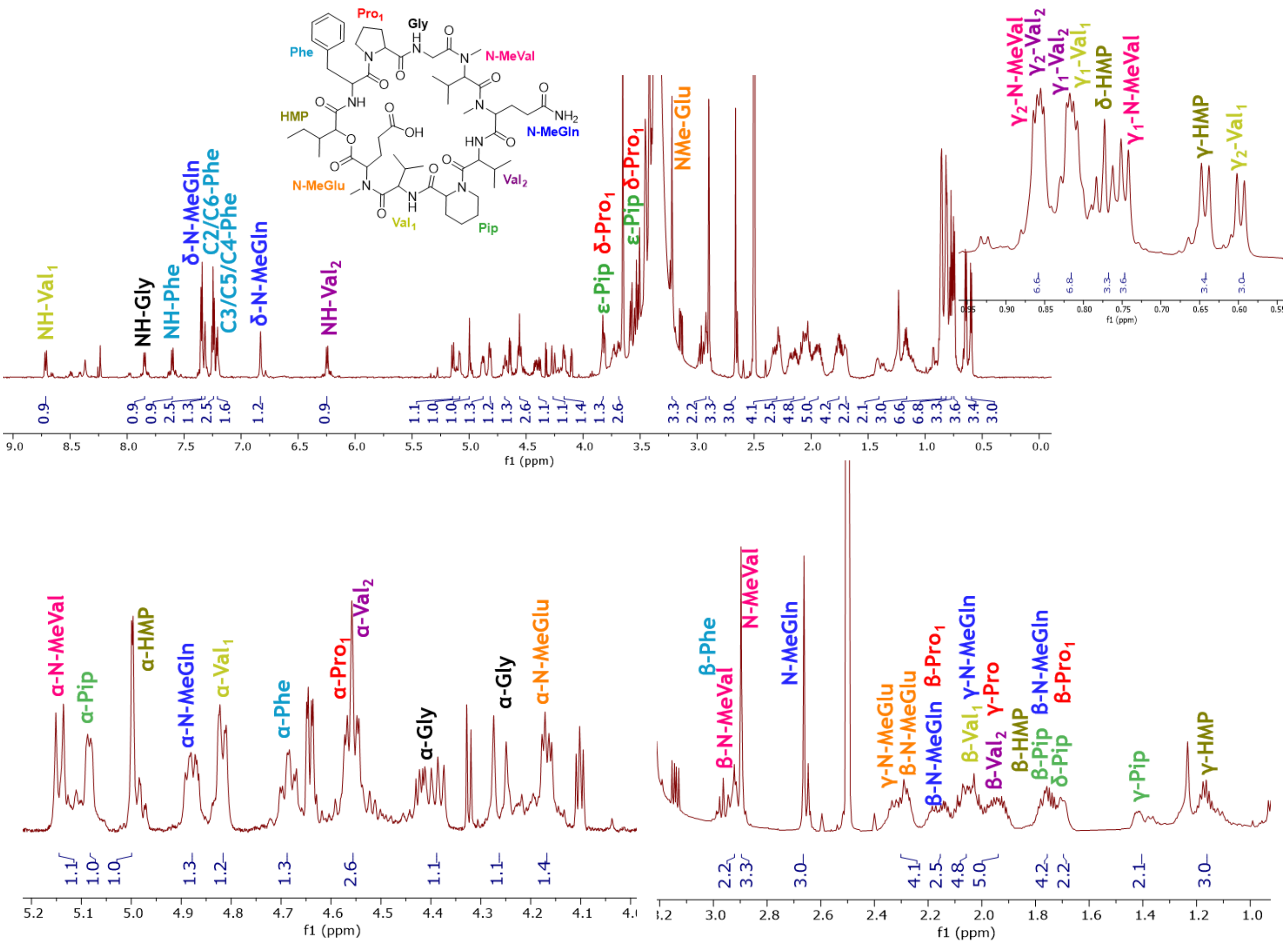

Figure S1. ^{1}H NMR spectrum of **1** in DMSO-d_6 (700 MHz).

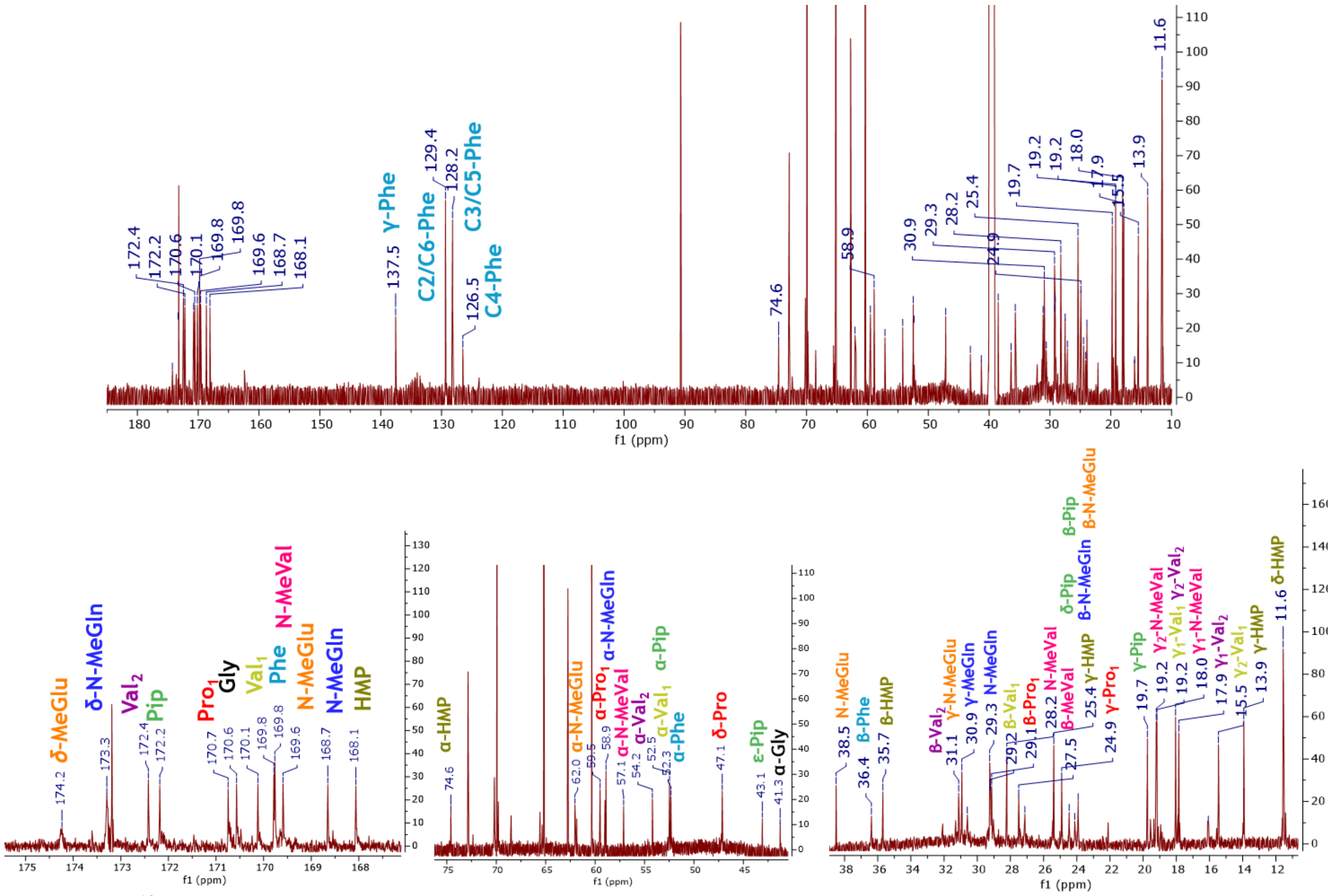

Figure S2. ^{13}C NMR spectrum of **1** in DMSO-d_6 (175 MHz).

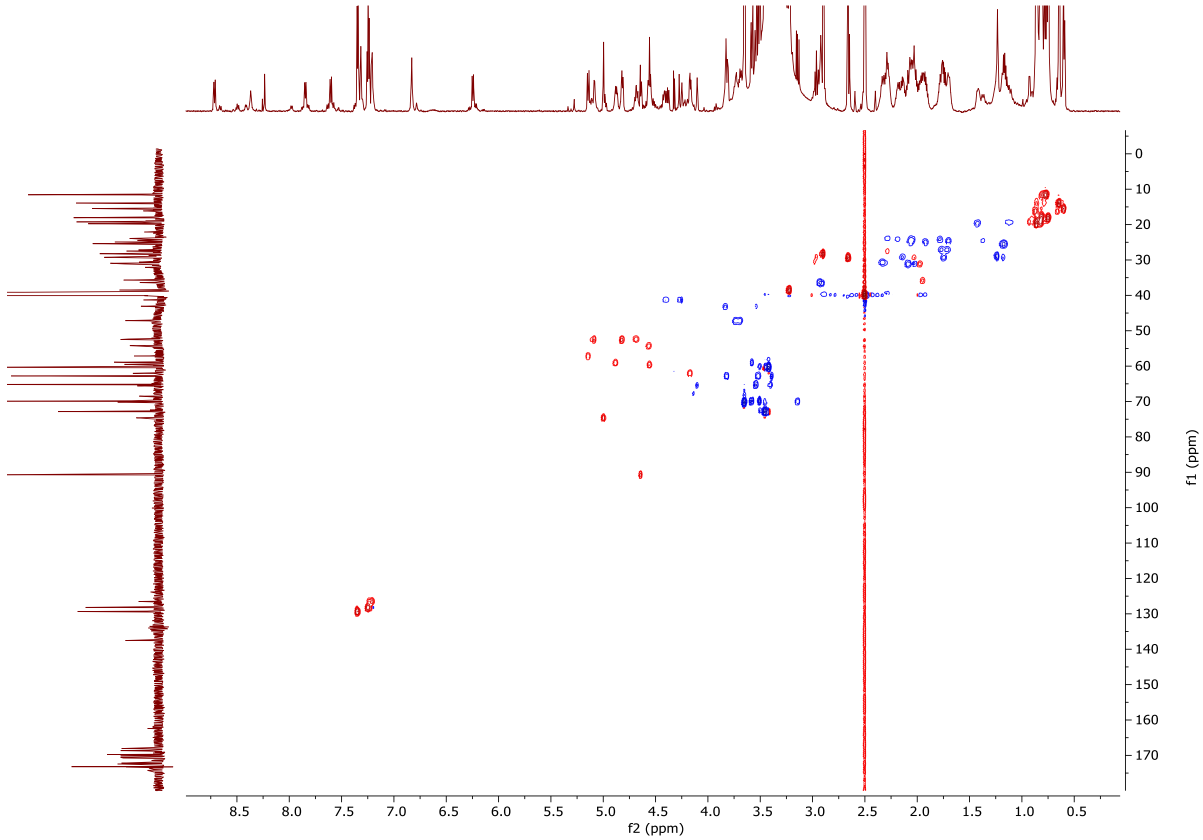

Figure S3. HSQC spectrum of **1** in DMSO-d_6 (700 MHz).

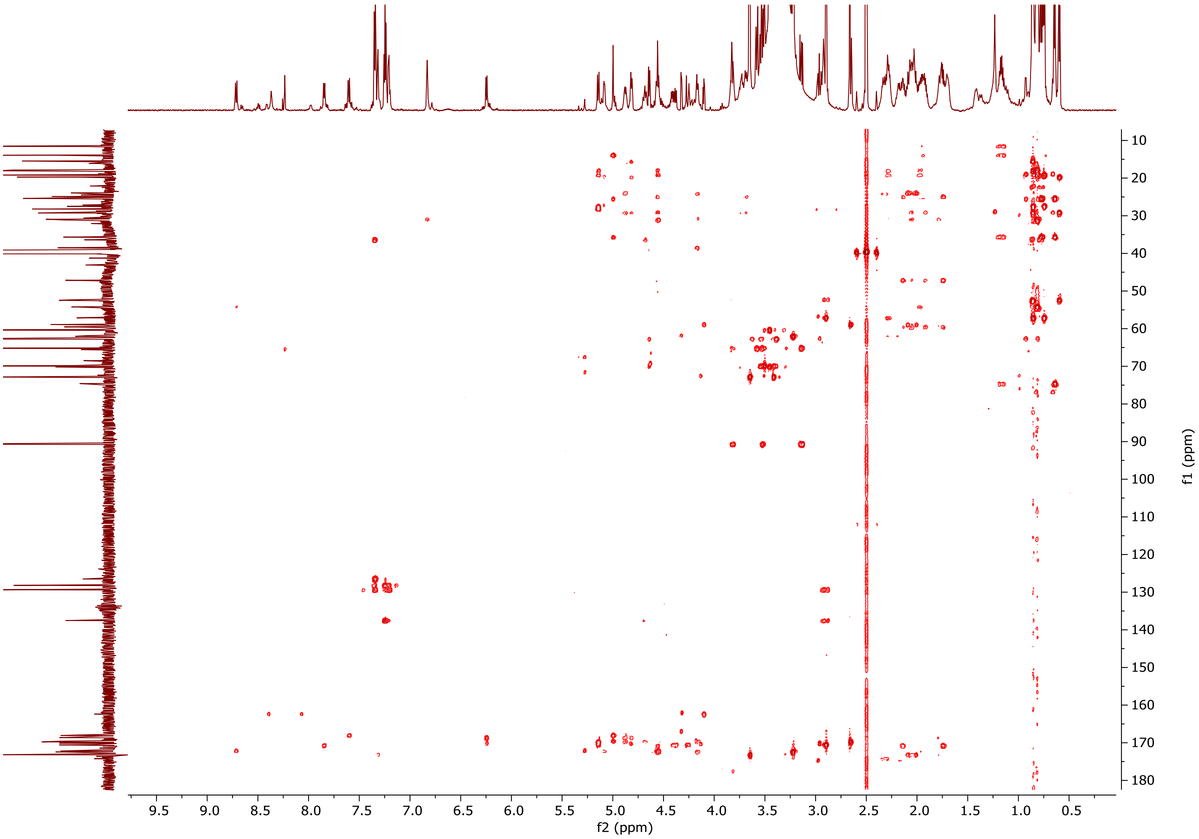

Figure S4. HMBC spectrum of **1** in DMSO-d_6 (700 MHz).

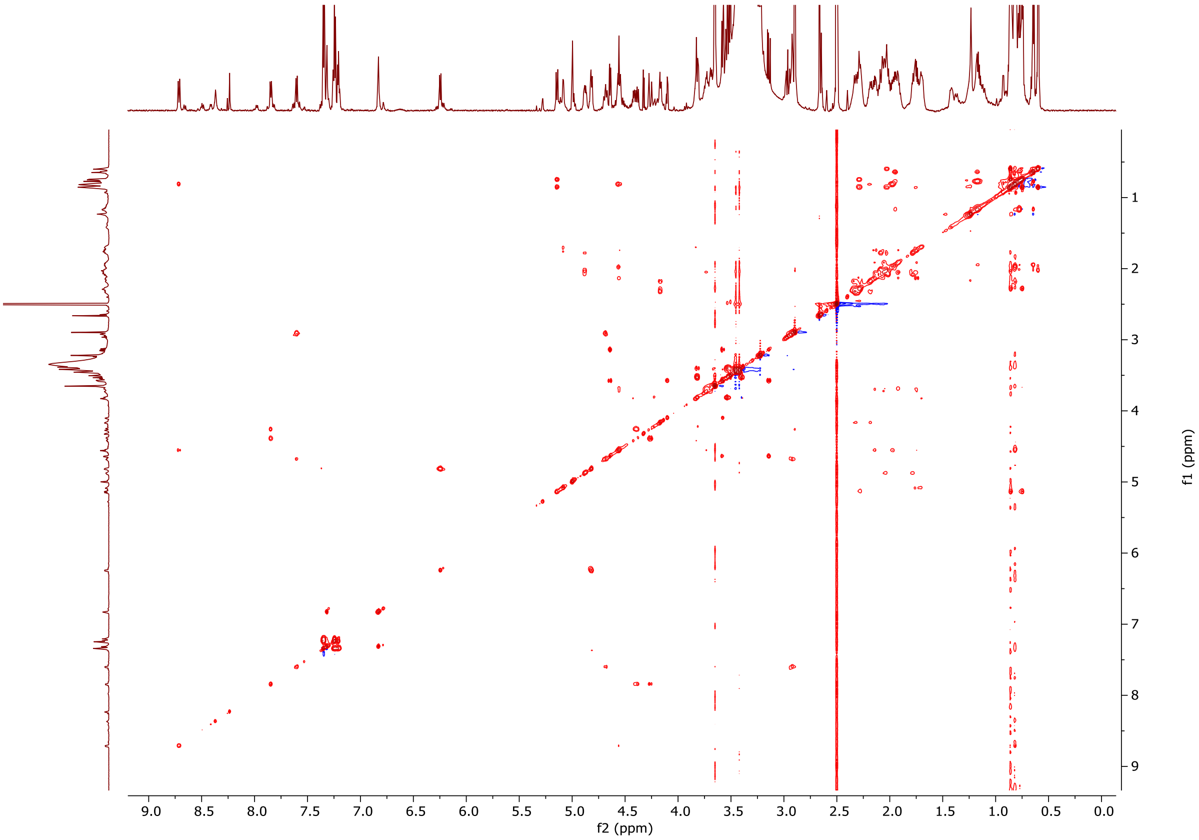

Figure S5. TOCSY spectrum of **1** in DMSO-d_6 (700 MHz).

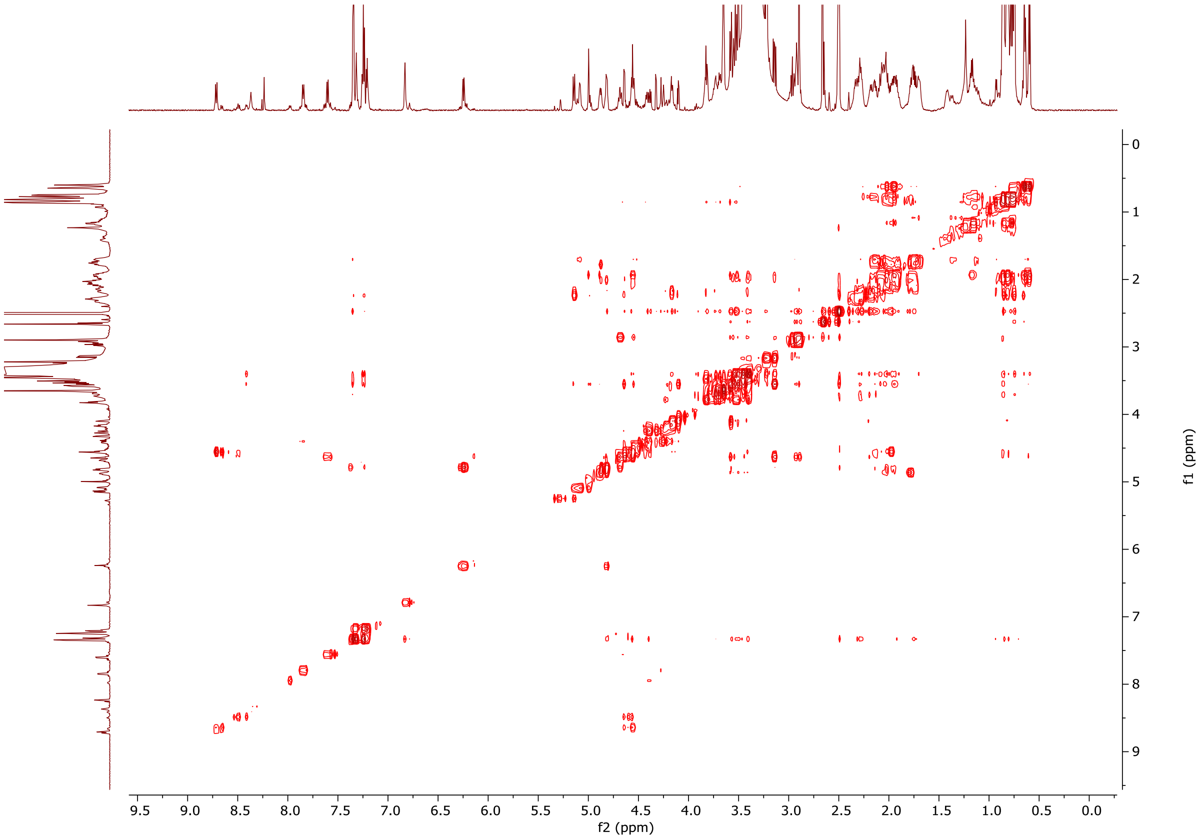

Figure S6. COSY spectrum of **1** in DMSO-d_6 (700 MHz).

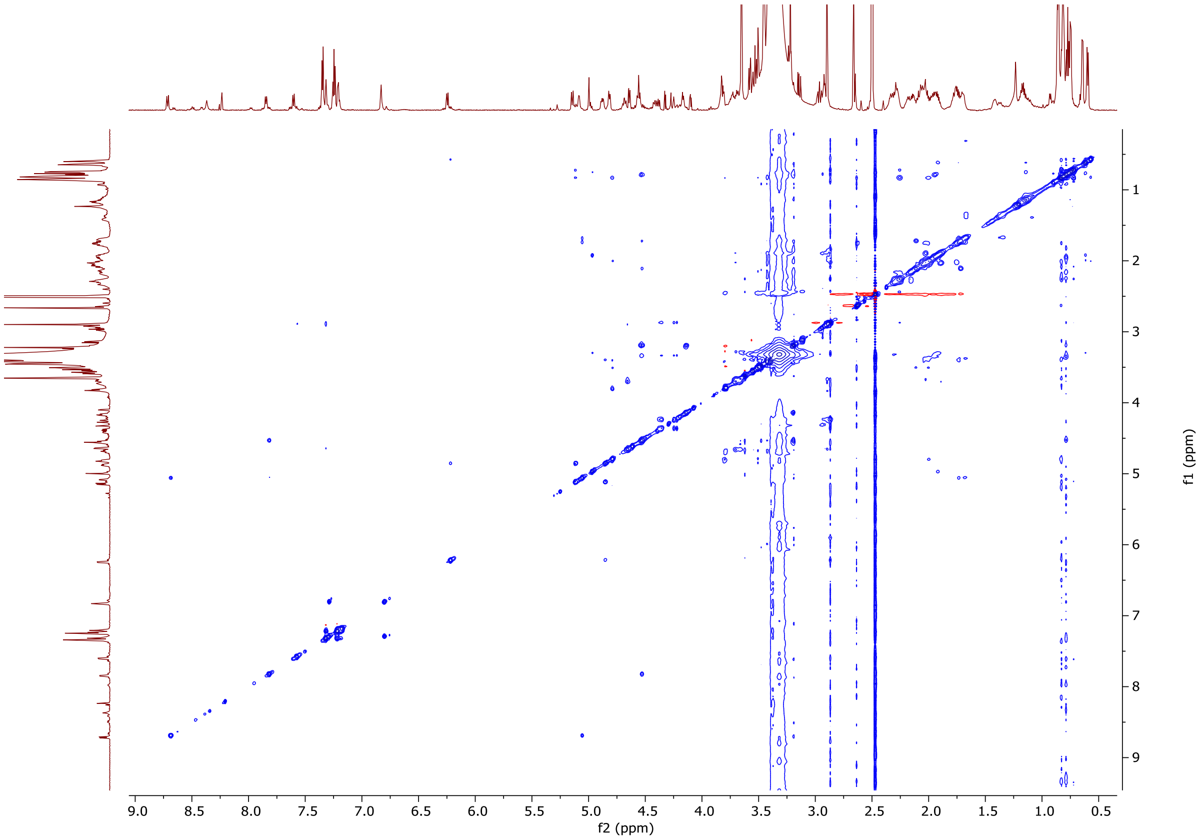

Figure S7. NOESY spectrum of **1** in DMSO-d_6 (700 MHz).

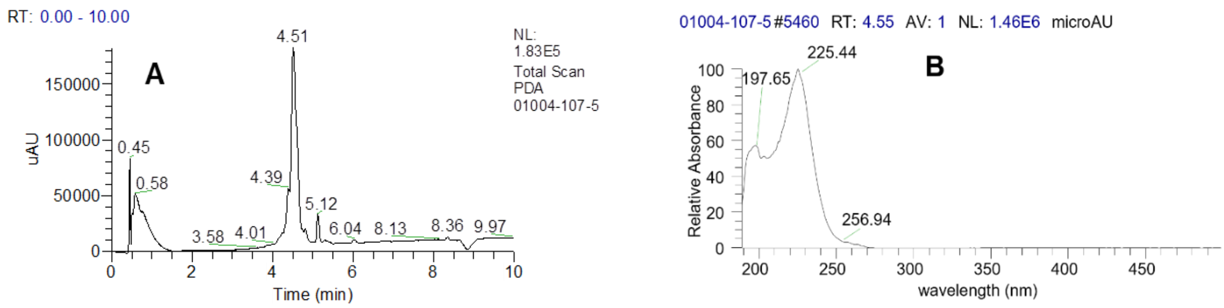

Figure S8. UPLC-PDA (λ 227 nm) of **1**.

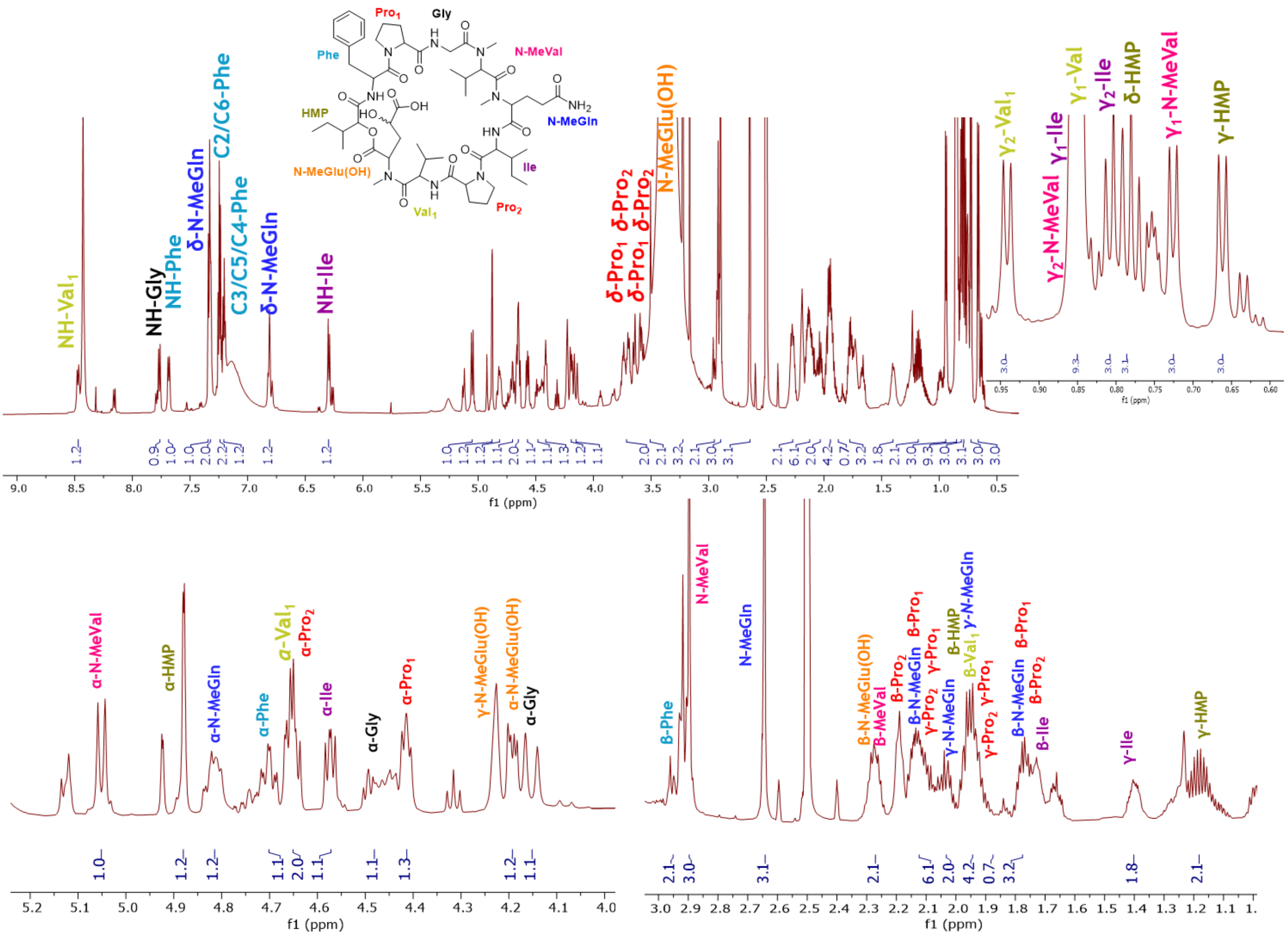

Figure S9. ^{1}H NMR spectrum of **2** in DMSO-d_6 (700 MHz).

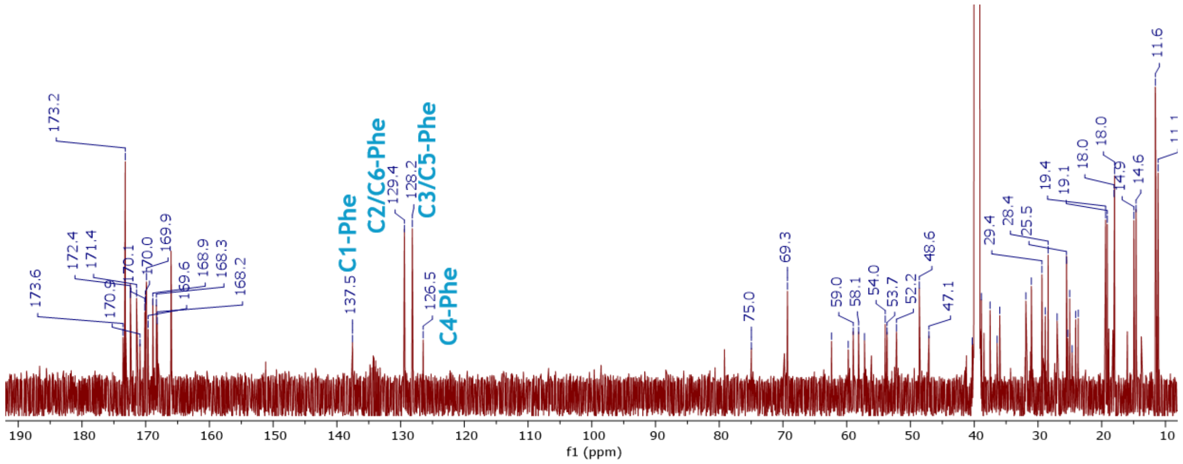

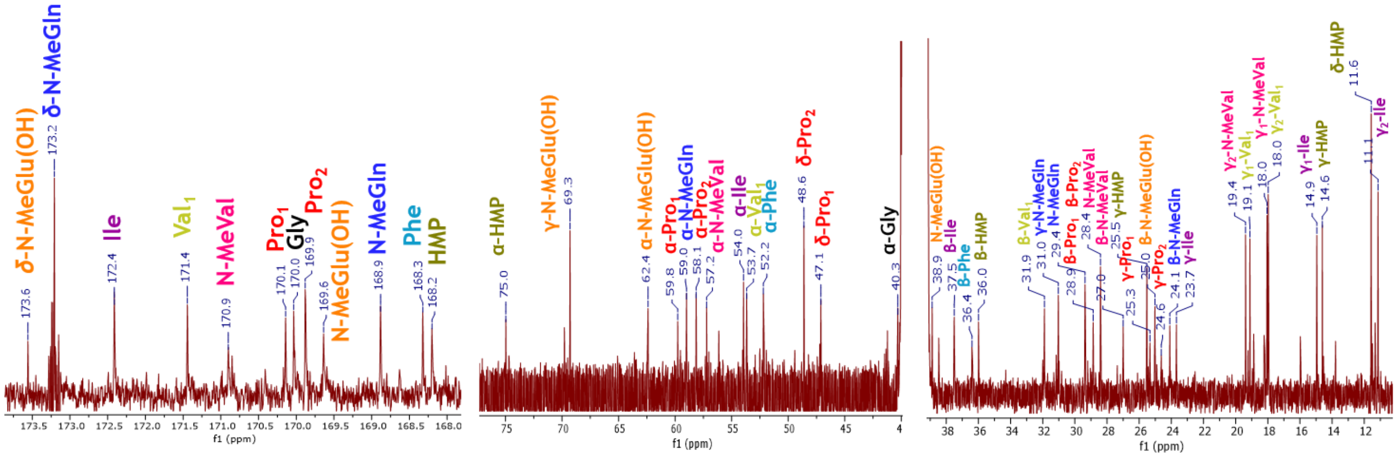

Figure S10. ^{13}C NMR spectrum of **2** in DMSO-d_6 (175 MHz).

A

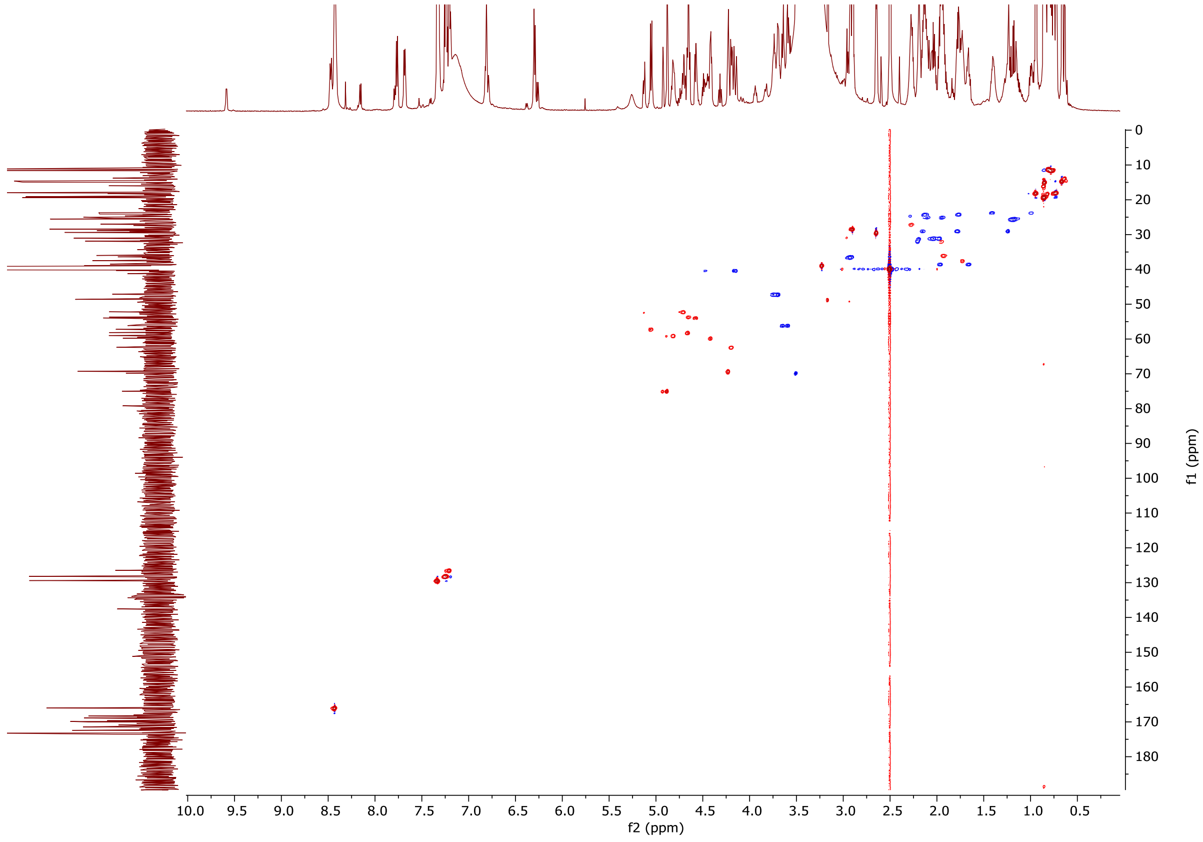

B

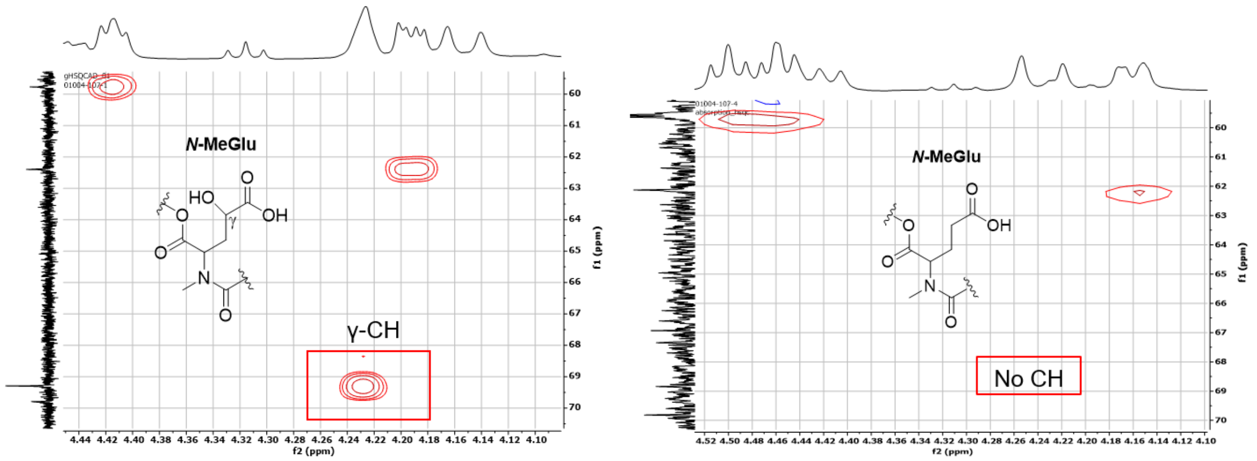

Figure S11. HSQC spectrum of **2** in DMSO-d_6 (700 MHz). (A) Full spectrum; (B) Comparison of region of addition CH in *N*-MeGlu of **2** (left) and **5** (right).

A

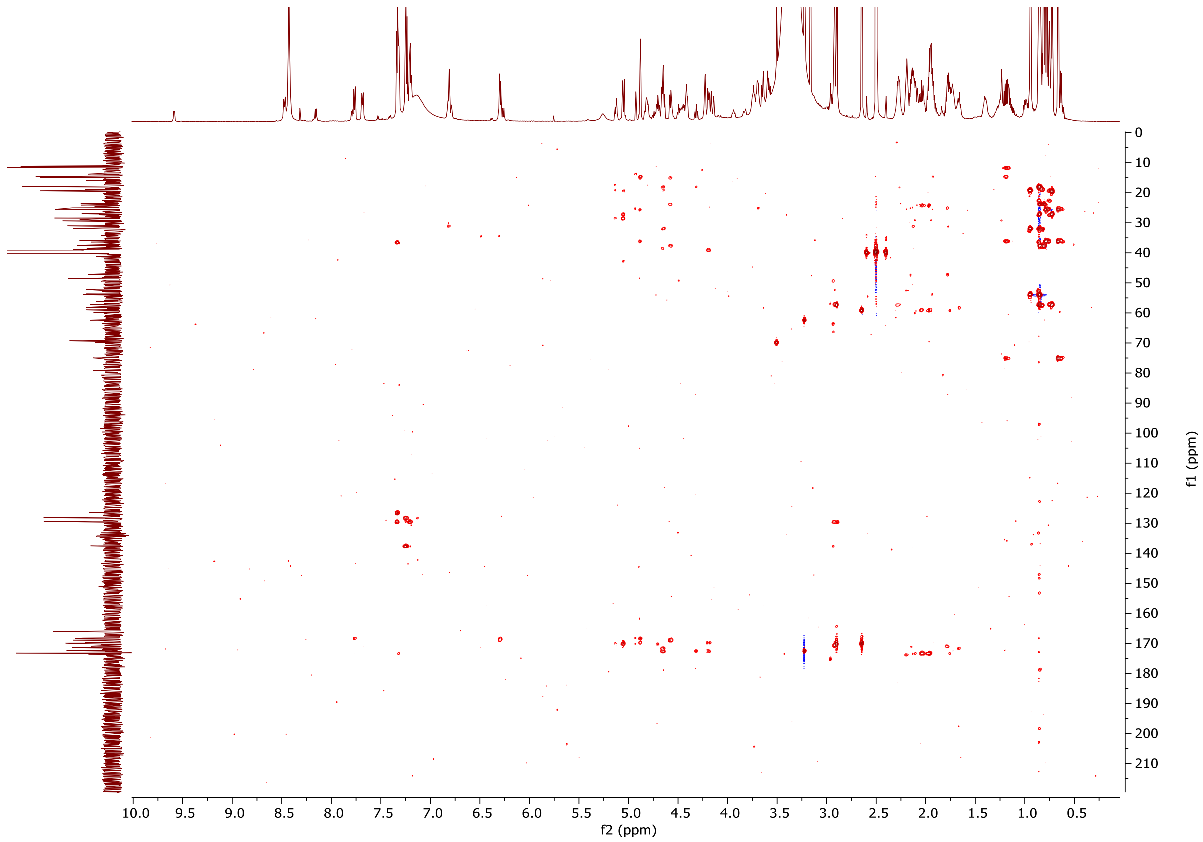

B

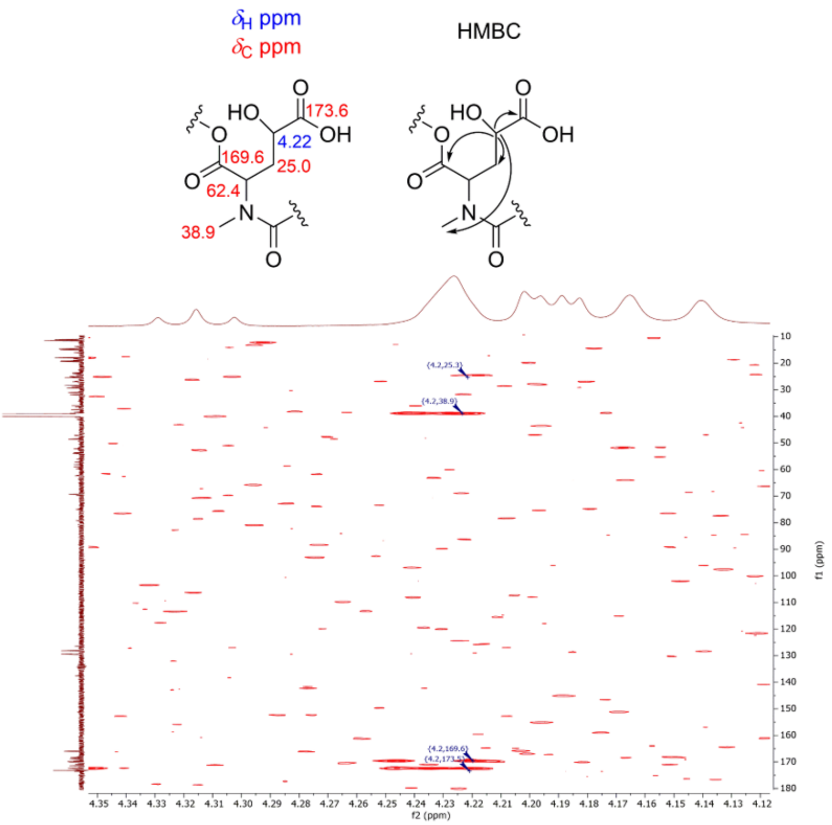

Figure S12. HMBC spectrum of **2** in DMSO-d_6 (700 MHz). (A) Full spectrum; (B) Key correlations in *N*-MeGlu.

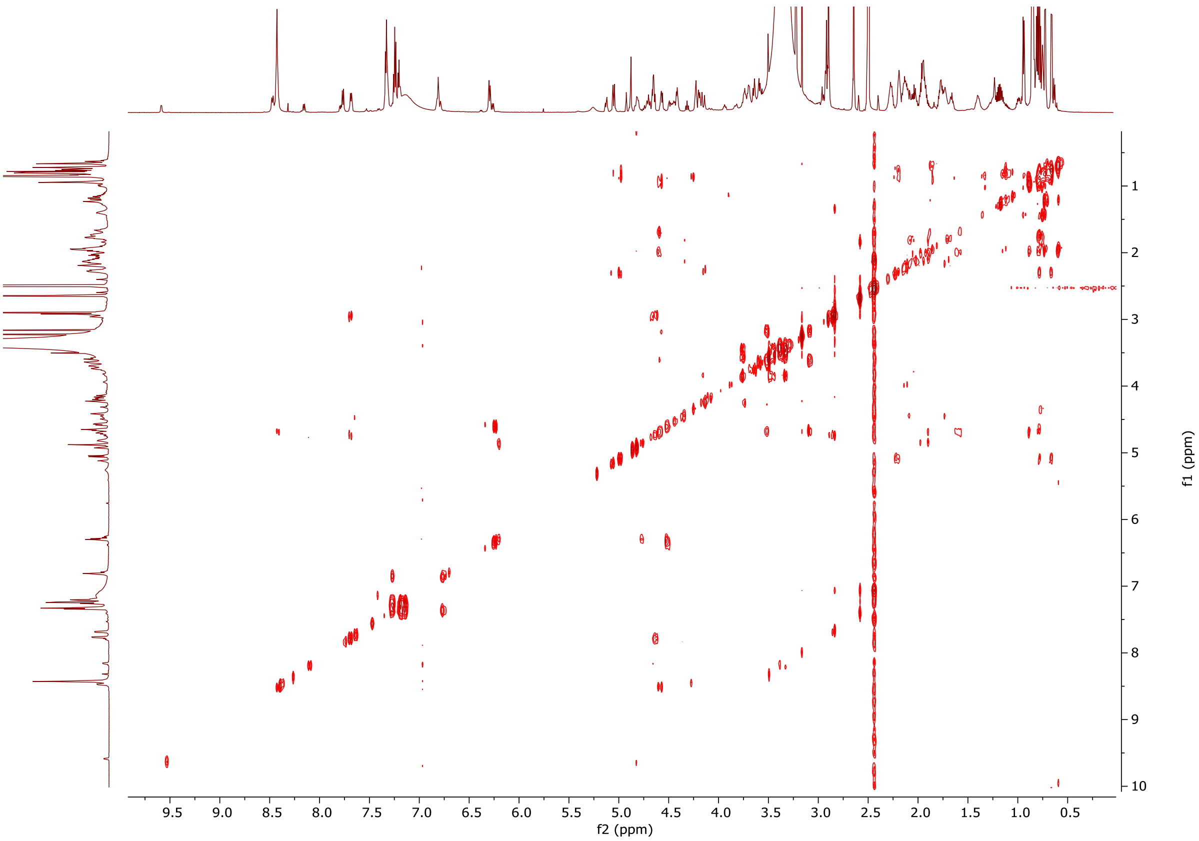

Figure S13. TOCSY spectrum of **2** in DMSO-d_6 (700 MHz).

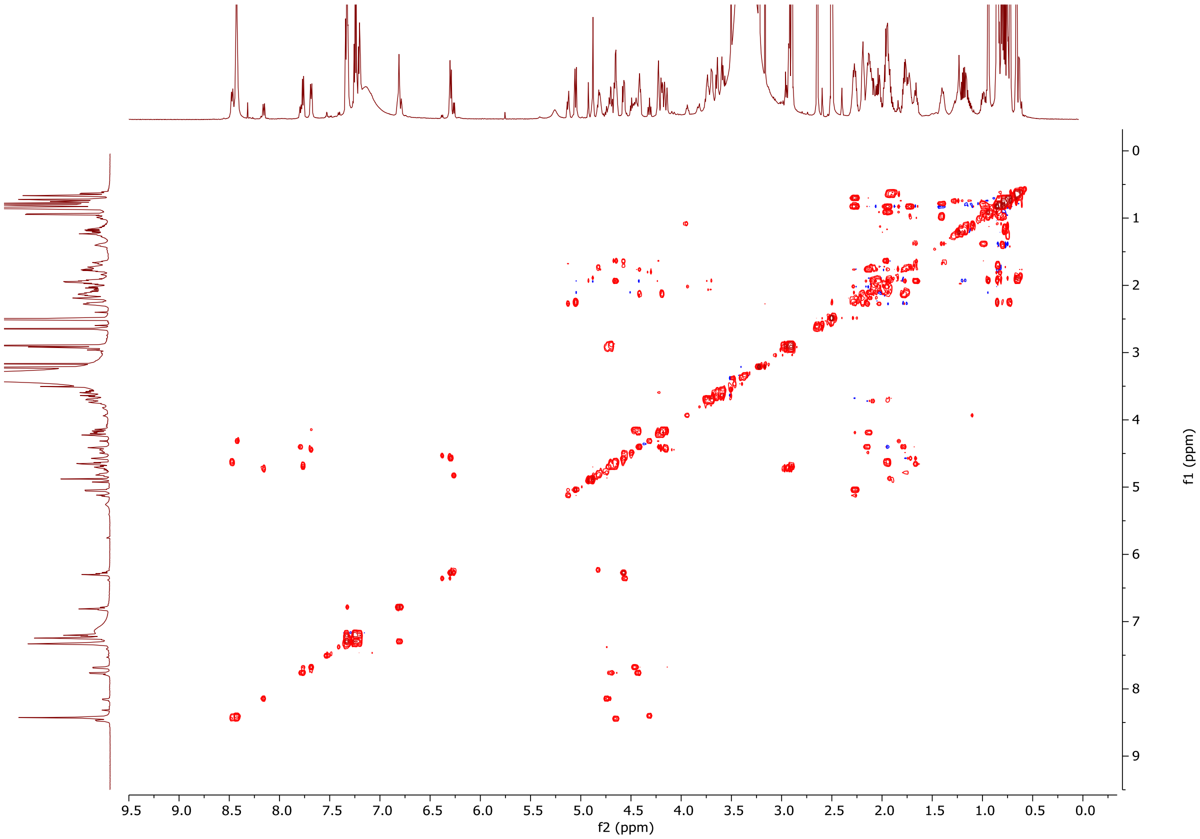

Figure S14. COSY spectrum of **2** in DMSO-d_6 (700 MHz).

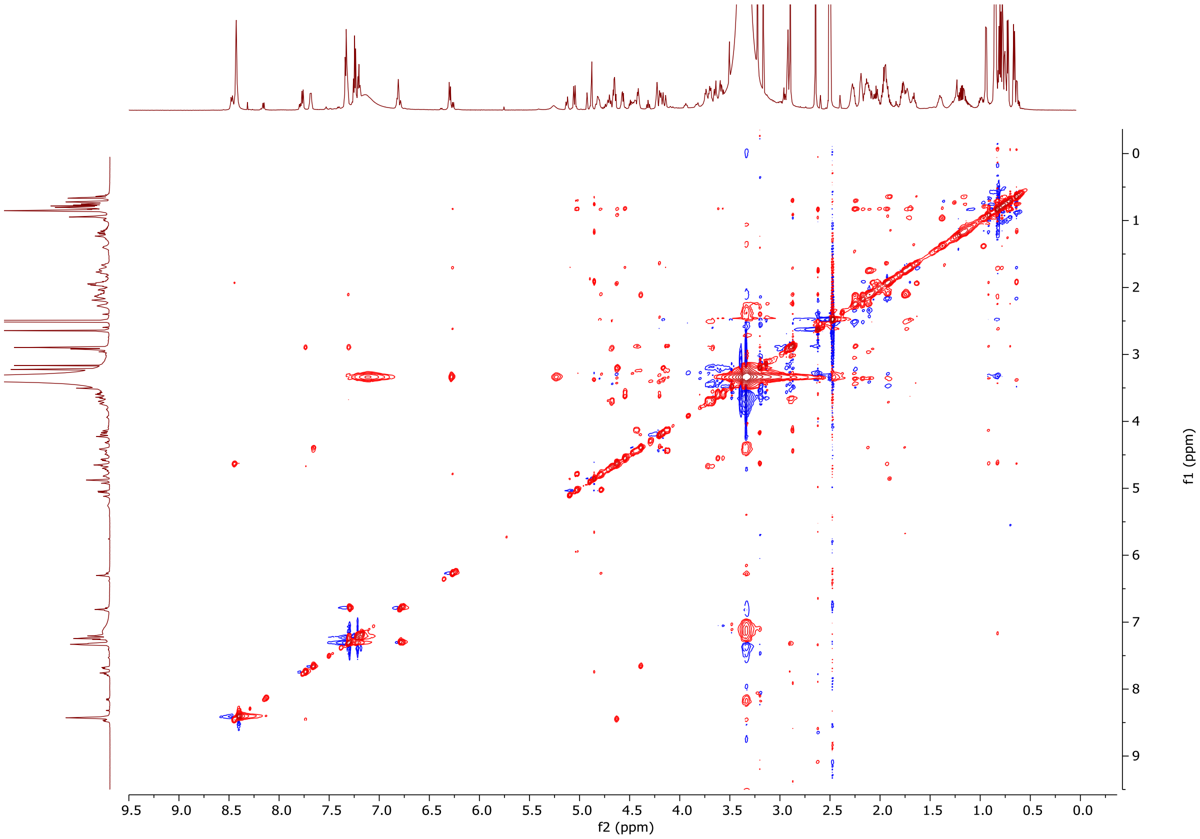

Figure S15. NOESY spectrum of **2** in DMSO-d_6 (700 MHz).

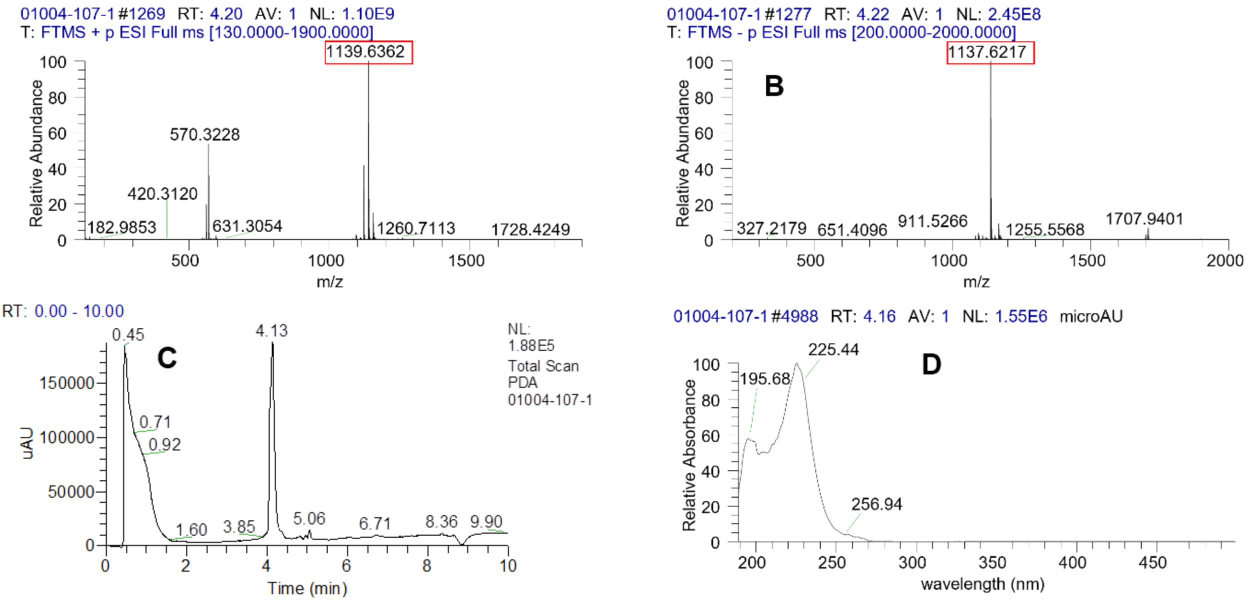

Figure S16. UPLC-PDA-HRESIMS of **2**: (A) ESI+; (B) ESI-; (C, D) PDA (λ 227 nm).

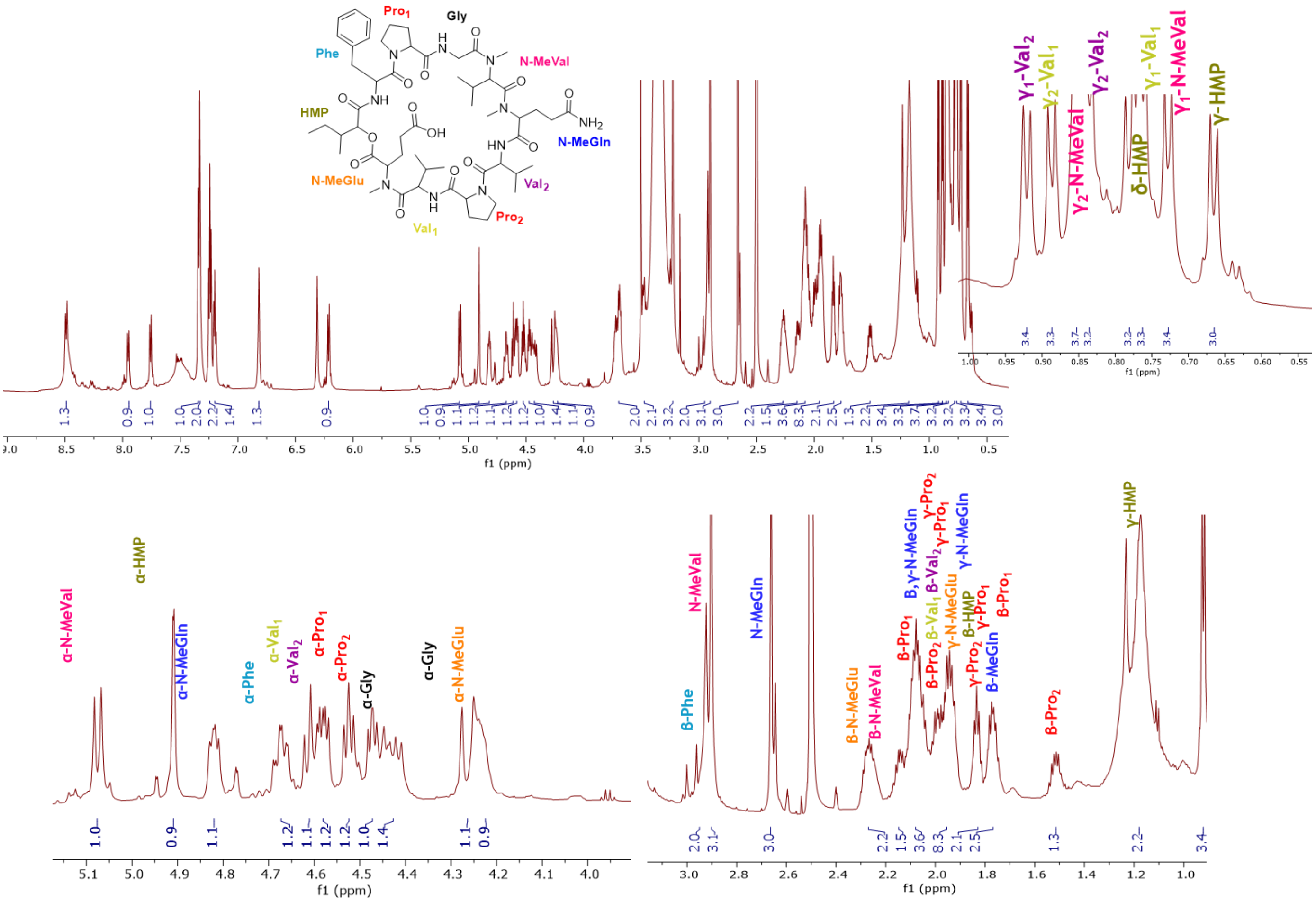

Figure S17. ^{1}H NMR spectrum of **3** in DMSO-d_6 (700 MHz).

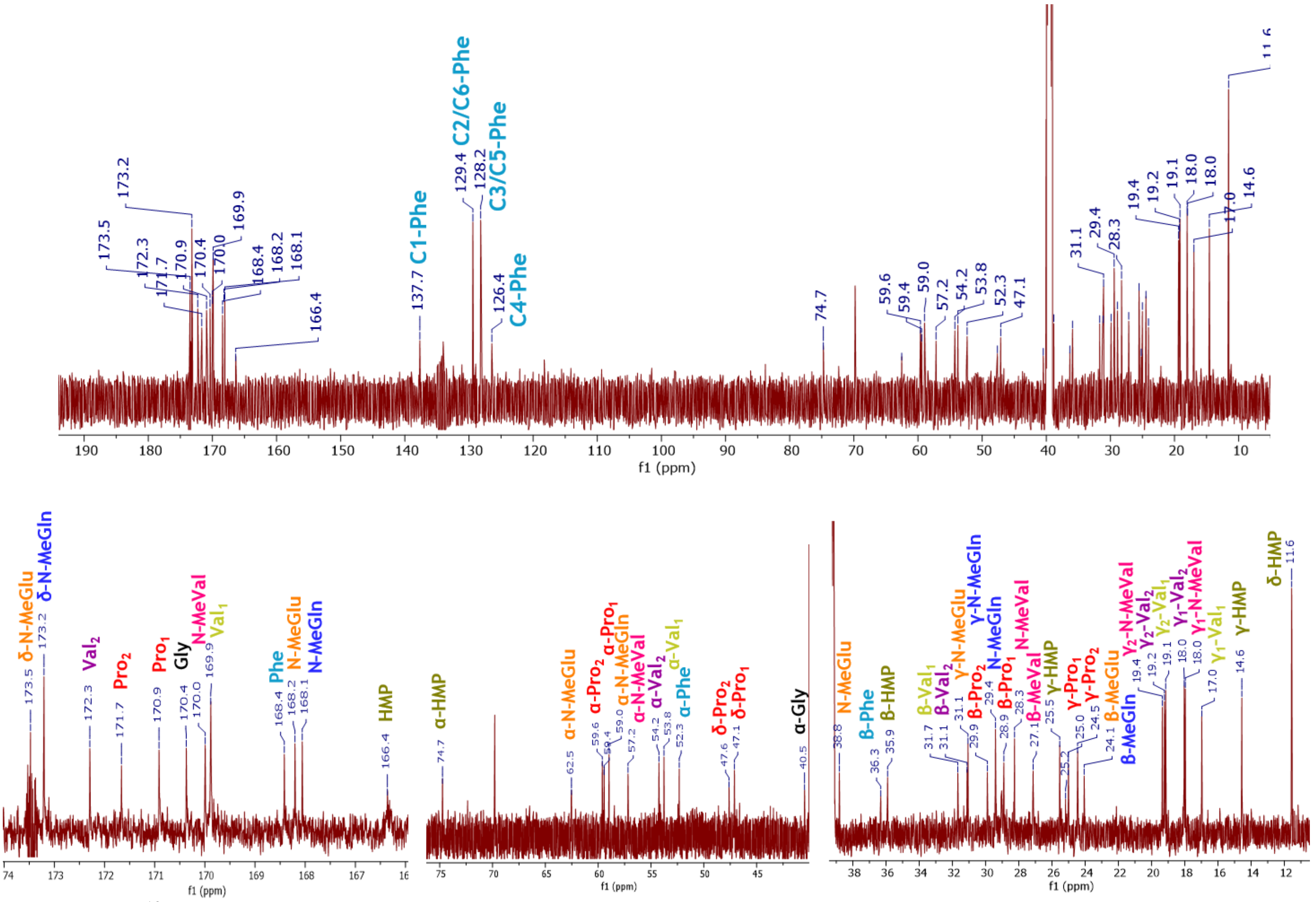

Figure S18. ^{13}C NMR spectrum of **3** in DMSO-d_6 (175 MHz).

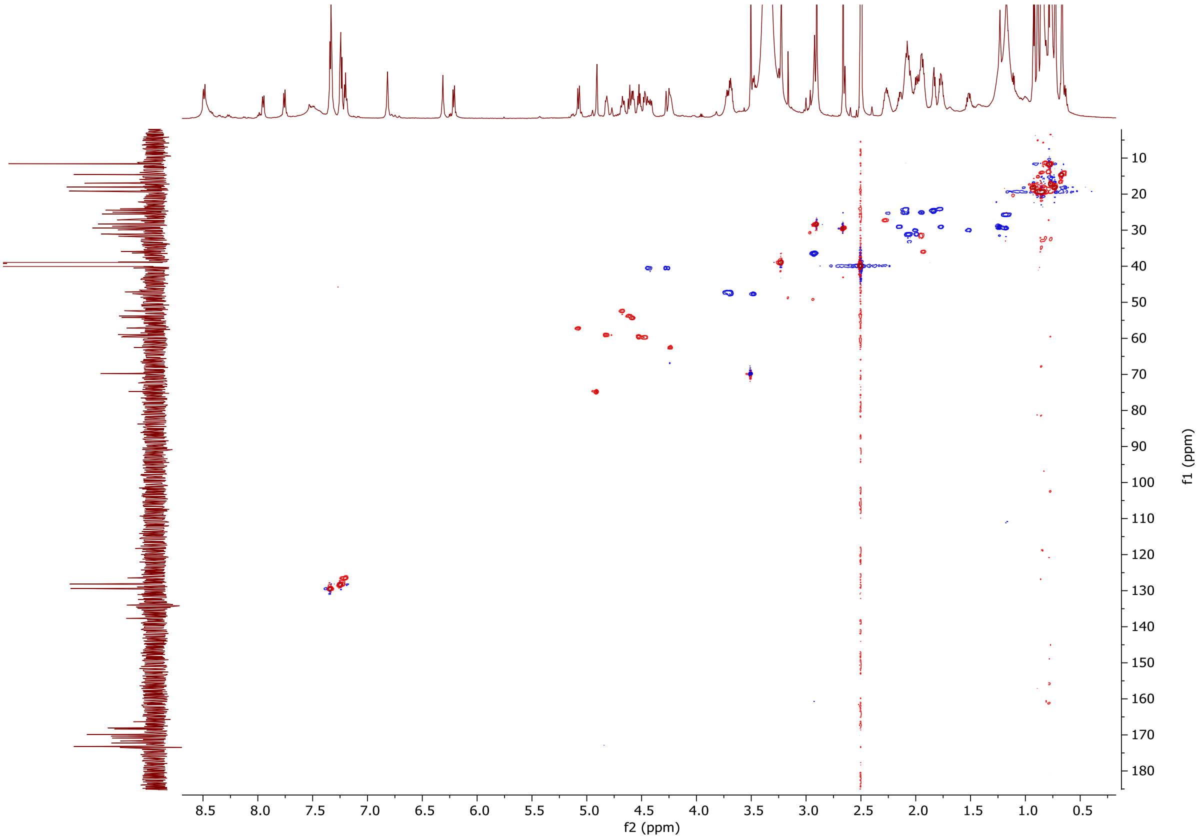

Figure S19. HSQC spectrum of **3** in DMSO-d_6 (700 MHz).

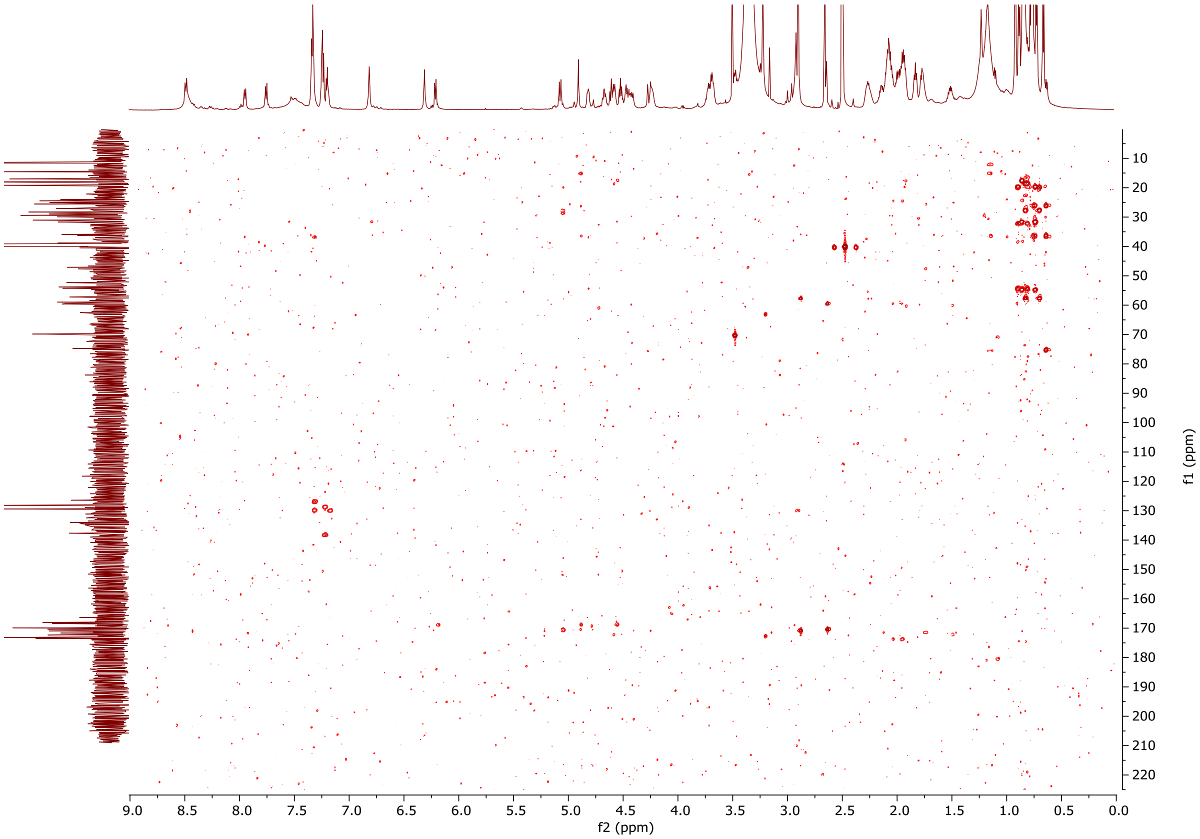

Figure S20. HMBC spectrum of **3** in DMSO-d_6 (700 MHz).

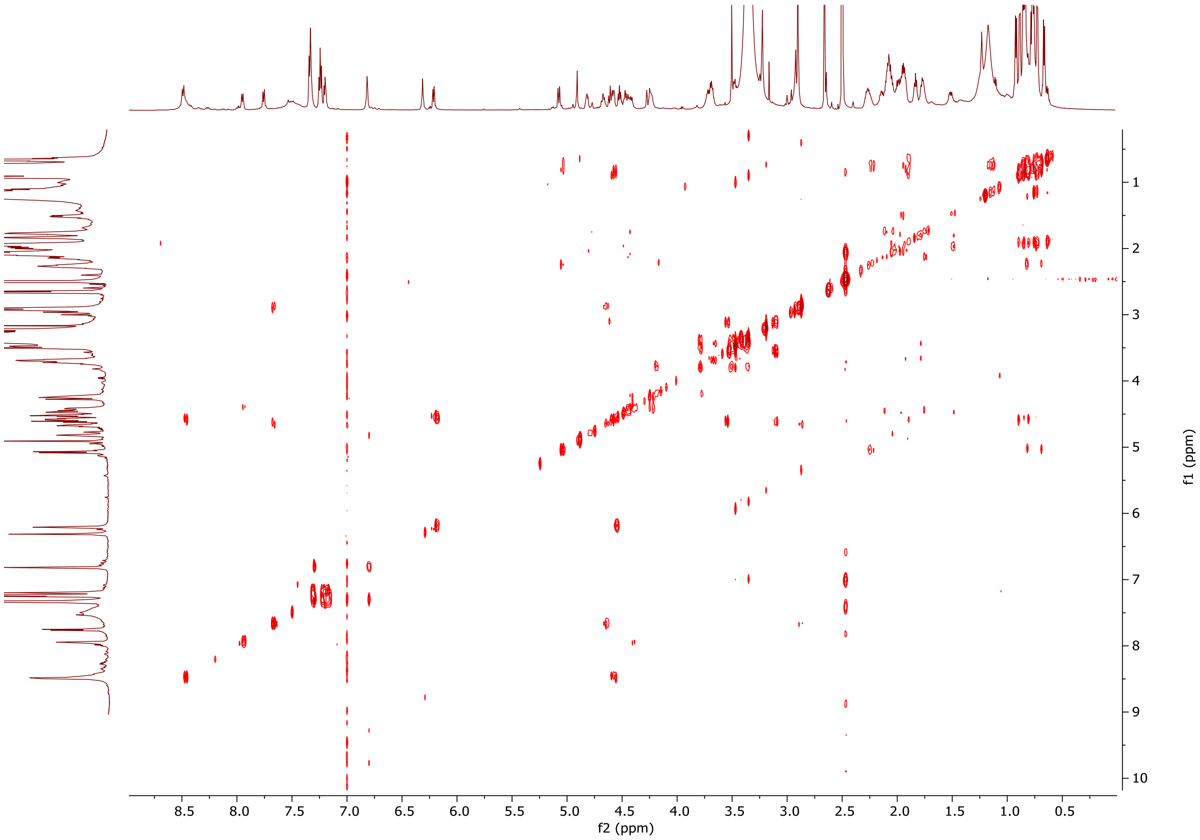

Figure S21. TOCSY spectrum of **3** in DMSO-d_6 (700 MHz).

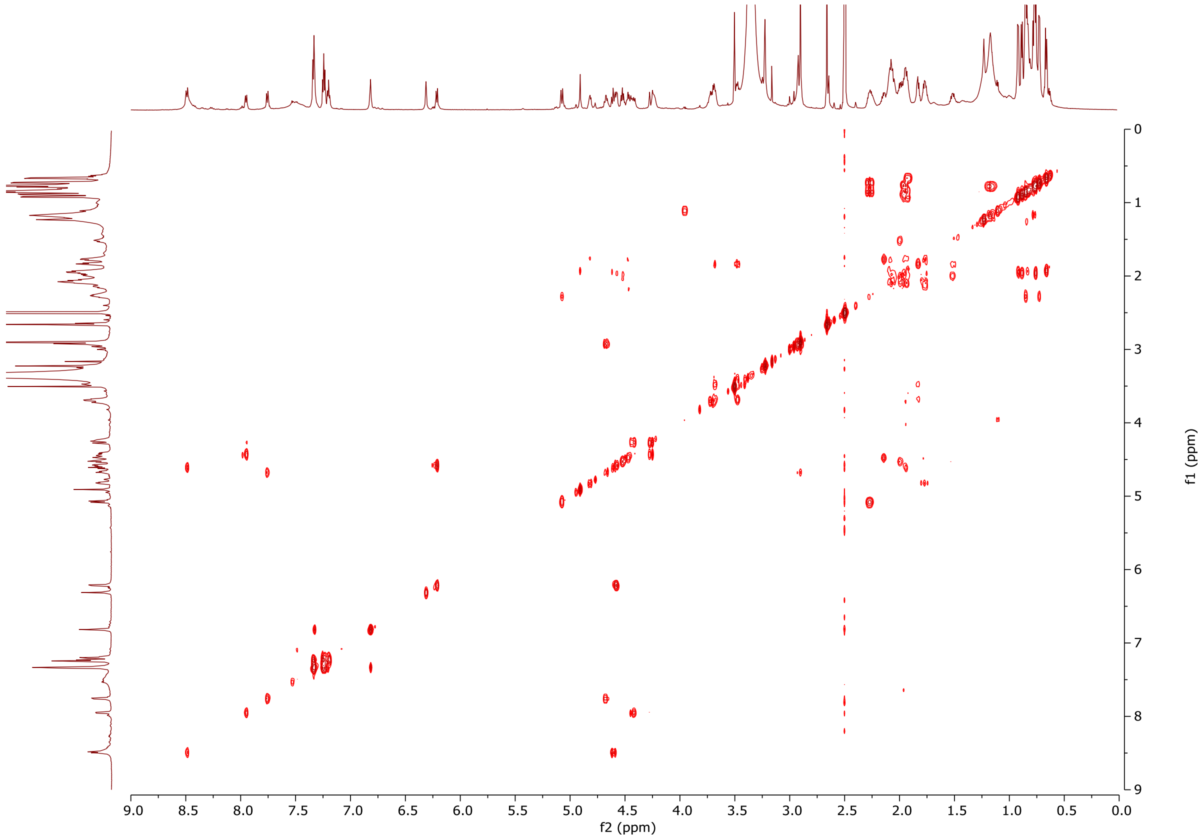

Figure S22. COSY spectrum of **3** in DMSO-d_6 (700 MHz).

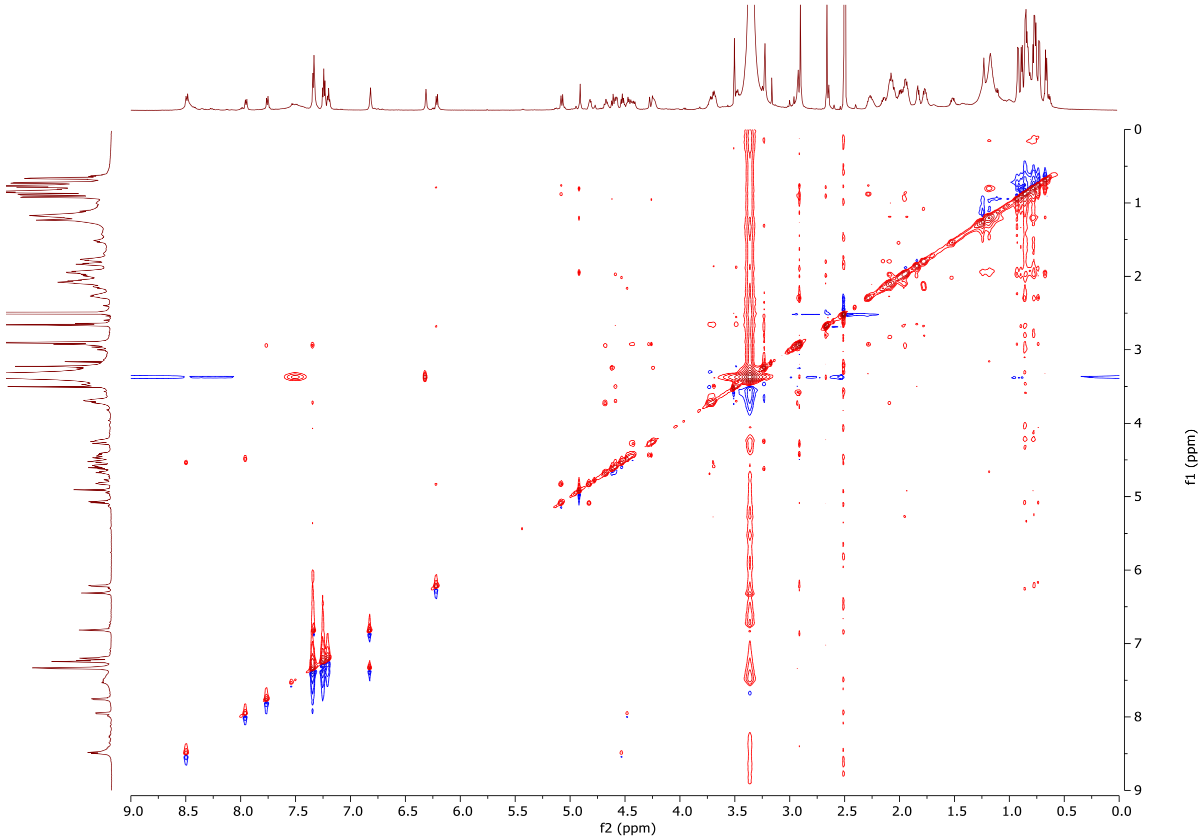

Figure S23. NOESY spectrum of **3** in DMSO-d_6 (700 MHz).

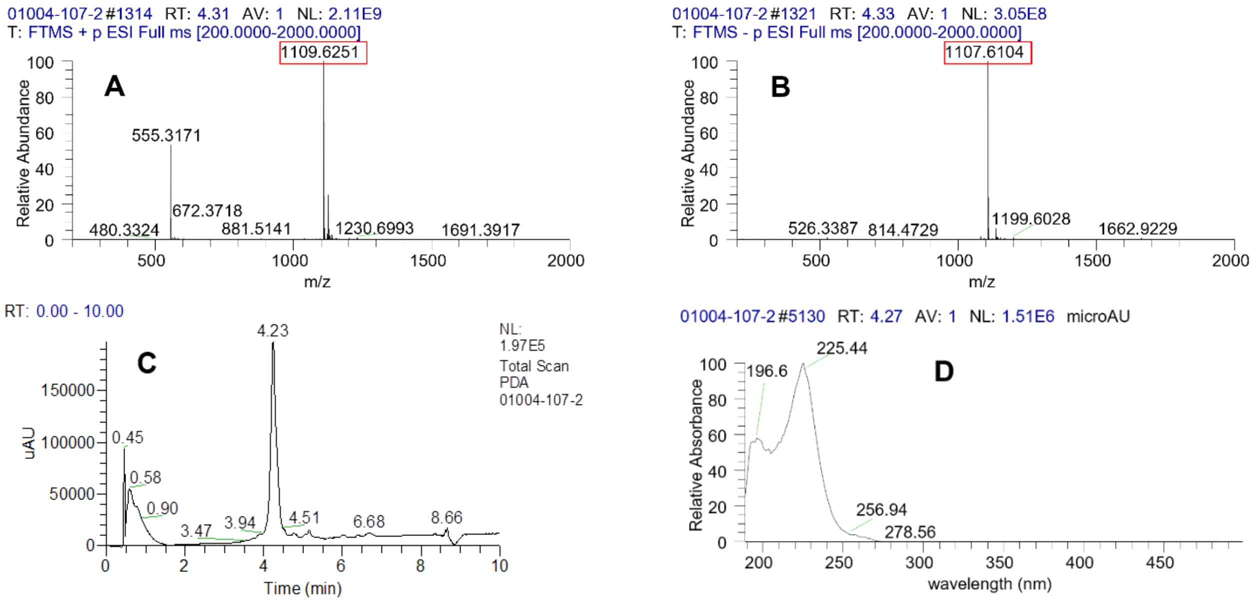

Figure S24. UPLC-PDA-HRESIMS of **3**: (A) ESI+; (B) ESI-; (C,D) PDA (λ 227 nm).

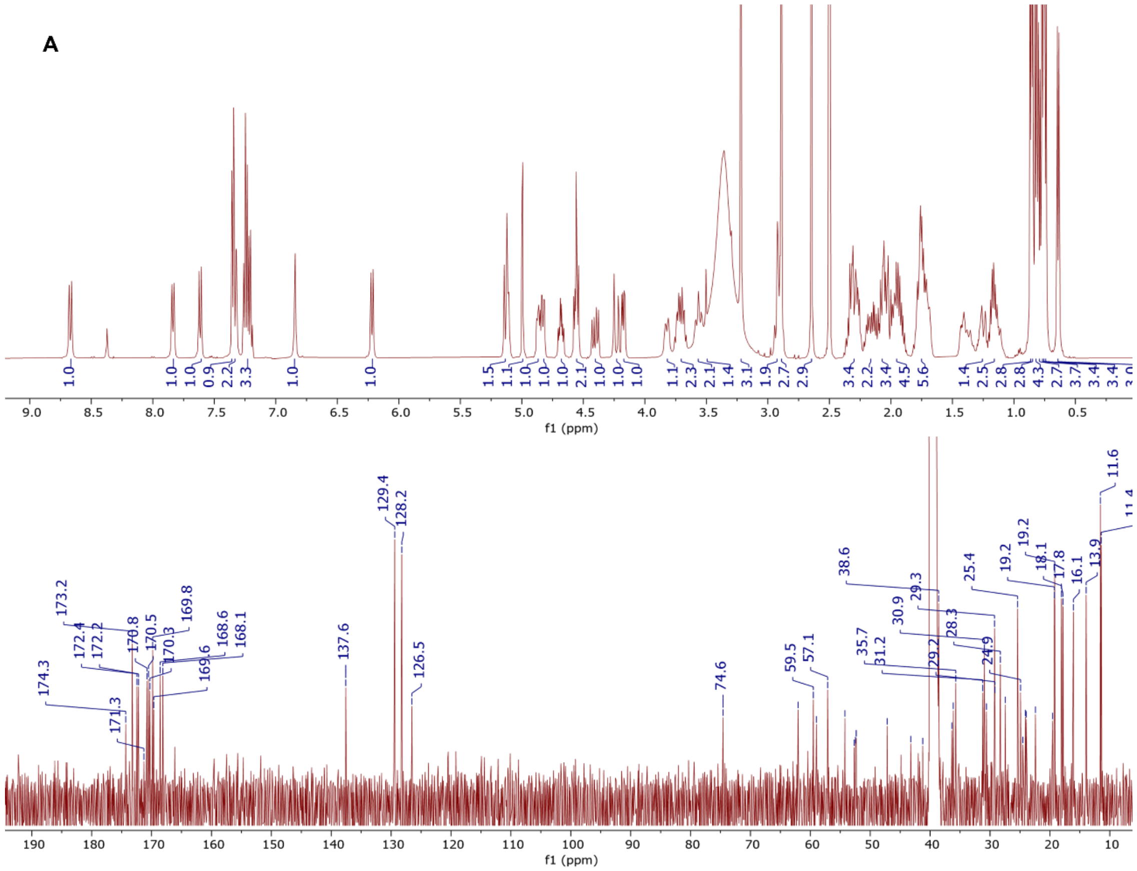

Figure S25. (A) ^{1}H and (B) ^{13}C NMR spectra of **4** in DMSO-d_6 (500 and 125 MHz, respectively).

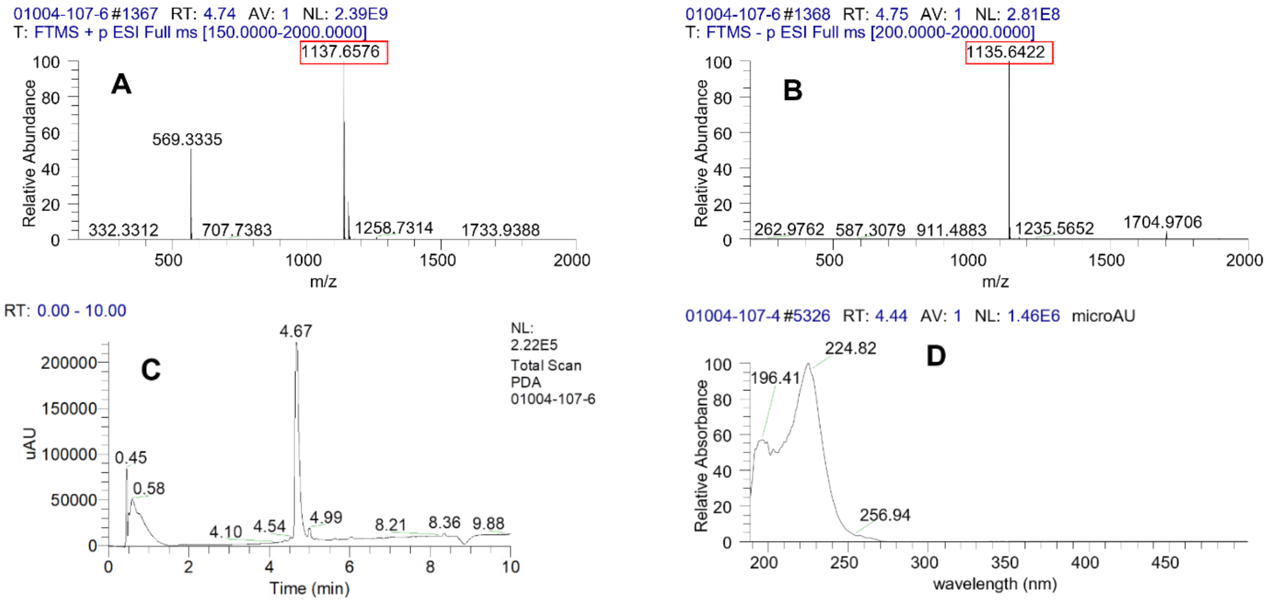

Figure S26. UPLC-PDA-HRESIMS of **4**: (**A**) ESI+; (**B**) ESI-; (**C, D**) PDA (λ 227 nm).

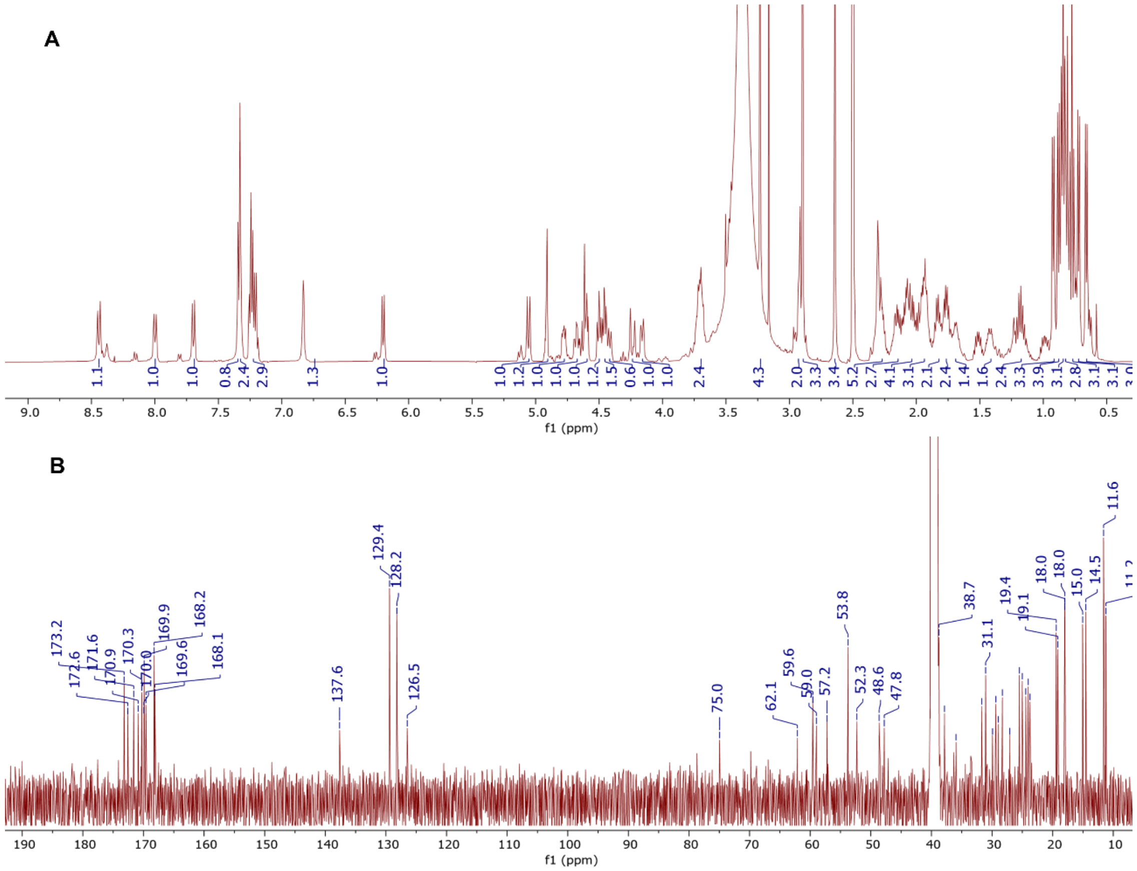

Figure S27. (A) ^{1}H and (B) ^{13}C NMR spectra of **5** in DMSO-d_6 (500 and 125 MHz, respectively).

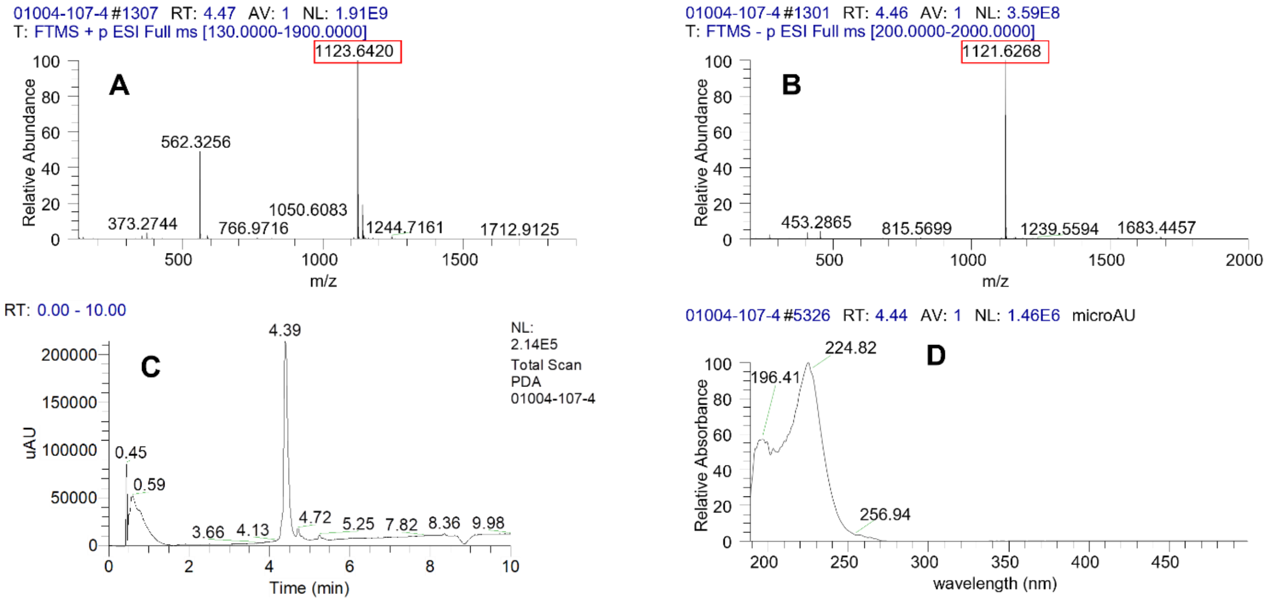

Figure S28. UPLC-PDA-HRESIMS of **5**: (A) ESI+; (B) ESI-; (C, D) PDA (λ 227 nm).

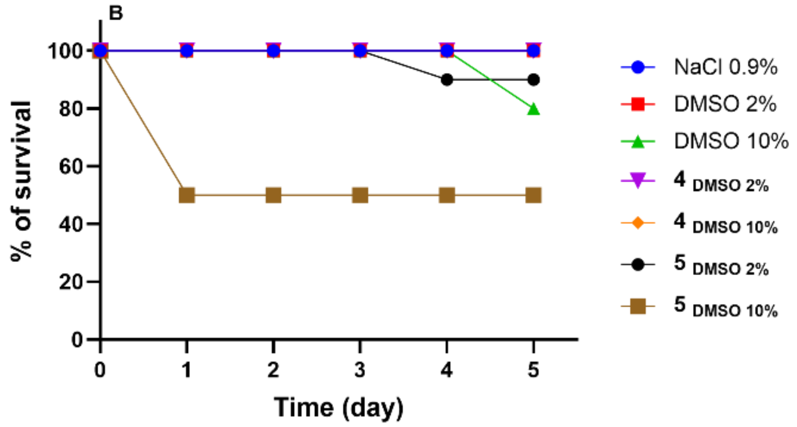

Figure S29. Toxicity results for the vehicles NaCl 0.9%, DMSO 2% and 10%, and **4** and **5** on *G. mellonella* larvae.

Table S1. ^{1}H and ^{13}C NMR Data for **4** and **5** in DMSO-d_6 (500 MHz and 125 MHz, respectively) [δ_H, mult. (J in Hz) in ppm; δ_C in ppm].

amino acid		4		5		amino acid		4		5	
***N*-MeGlu**	CO		169.6		169.6	***N*-MeVal**	CO		169.8		169.9
	α	4.17, dd (9.6, 4.2)	62.0	4.16, d (9.2)	62.1		α	5.13, d (10.9)	57.1	5.06, d (10.5)	57.2
	β	2.29, m<br>2.16, m	24.1	2.26, m	24.0		β	2.29, m	27.4	2.26, m	27.1
	γ	2.29, m	30.6	2.30, m	31.1		γ$_1$-Me	0.74, d (6.7)	18.1	0.72, d (6.8)	18.0
	δ-COOH		174.3		173.2		γ$_2$-Me	0.82, d (6.7)	19.2	0.85, d (6.8)	19.1
	NMe	3.22, s	38.6	3.23, s	38.7		NMe	2.89, s	28.3	2.90, s	28.3
**Val$_1$**	CO		172.4		171.6	**Gly**	CO		171.3		170.3
	α	4.55, m	54.2	4.59, m	53.8		α	4.40, dd (17.8, 8.5)<br>4.23, d (17.8)	41.2	4.41, dd (16.5, 9.1)<br>4.23, d (16.5)	40.1
	β	1.95, m	31.2	1.93, m	31.7		NH	8.67, d (9.0)		8.0, d (9.0)	
	γ$_1$-Me	0.81, d (6.7)	17.8	0.92, d (6.8)	18.0	**Pro$_1$**	CO		172.2		170.9
	γ$_2$-Me	0.85, d (6.7)	19.2	0.84, d (6.8)	19.4		α	4.55, m	59.5	4.46, dd (8.0, 5.8)	59.6
	NH	8.66, d (9.6)		8.44, d (10.0)			β	2.16, m<br>1.75, m	29.2	2.14, m<br>1.50, m	29.9
**Pro$_2$/Pip**	CO		170.5		168.2		γ	2.05, m<br>1.95, m	24.9	1.83, m	24.4
	α	5.13, d (2.8)	52.5	4.52, t (7.2)	59.6		δ	3.71, m	47.2	3.46, m	48.6
	β	1.75, m	27.4	2.14, m<br>1.75, m	28.9	**Phe**	CO		170.3		169.9
	γ	1.40, m<br>1.16, m	19.6	2.05, m<br>1.93, m	25.0		α	4.68, td (9.0, 5.0)	52.3	4.67, td (9.4, 3.5)	52.3
	δ	1.75, m	24.5	3.70, m	47.8		β	2.92, m	36.4	2.92, m	36.2
	ε	3.82, m<br>3.56, m	43.2				γ-C$_1$		137.6		137.6
**Ile**	CO		170.8		170.0		C$_2$/C$_6$	7.34, m	129.4	7.33, m	129.4
	α	4.83, dd (8.7, 3.1)	52.7	4.62, d (9.7)	53.8		C$_3$/C$_5$	7.23, m	128.2	7.22, m	128.2
	β	1.75, m	36.1	1.68, m	37.8		C$_4$	77.23, m	126.5	7.22, m	126.5
	γ-Me	0.86, d (6.7)	16.1	0.88, d (6.8)	15.0		NH	7.61, d (8.9)		7.7, d (9.0)	
	γ-CH$_2$	1.26, m	22.4	1.42, m	23.7	**HMP**	CO		168.1		168.1
	δ-Me	0.76, d (6.7)	11.4	0.79, d (6.8)	11.2		α	4.99, d (2.0)	74.6	4.91, d (2.0)	75.0
	NH	6.21, d (8.5)		6.20, d (8.5)			β	1.95, m	35.7	1.93, m	35.9
***N*-MeGln**	CO		168.6		168.2		γ-Me	0.64, d (6.7)	13.9	0.66, d (6.8)	14.5
	α	4.86, dd (8.3, 5.3)	59.0	4.78, dd (9.0, 5.1)	59.0		γ-CH$_2$	1.16, m	25.4	1.18, m	25.5
	β	2.05, m<br>1.75, m	23.9	2.05, m<br>1.75, m	24.0		δ-Me	0.76, d (6.7)	11.6	0.77, d (6.8)	11.6
	γ	2.05, m<br>1.95, m	30.9	2.05, m<br>1.93, m	31.1						
	δ-CONH$_2$	7.35, bs<br>6.84, bs	173.2	7.35, bs<br>6.83, bs	172.6						
	NMe	2.64, s	29.3	2.64, s	29.4						
